# Systematic in-silico evaluation of fibrosis effects on re-entrant wave dynamics in atrial tissue

**DOI:** 10.1038/s41598-024-62002-5

**Published:** 2024-05-19

**Authors:** Michela Masè, Alessandro Cristoforetti, Samuele Pelloni, Flavia Ravelli

**Affiliations:** 1 Laboratory of Biophysics and Translational Cardiology, Department of Cellular, Computational and Integrative Biology—CIBIO, University of Trento, Via Sommarive 18, 38123 Povo, Trento, Italy; 2 CISMed—Centre for Medical Sciences, University of Trento, 38122 Trento, Italy

**Keywords:** Cardiology, Computational biophysics

## Abstract

Despite the key role of fibrosis in atrial fibrillation (AF), the effects of different spatial distributions and textures of fibrosis on wave propagation mechanisms in AF are not fully understood. To clarify these aspects, we performed a systematic computational study to assess fibrosis effects on the characteristics and stability of re-entrant waves in electrically-remodelled atrial tissues. A stochastic algorithm, which generated fibrotic distributions with controlled overall amount, average size, and orientation of fibrosis elements, was implemented on a monolayer spheric atrial model. 245 simulations were run at changing fibrosis parameters. The emerging propagation patterns were quantified in terms of rate, regularity, and coupling by frequency-domain analysis of correspondent synthetic bipolar electrograms. At the increase of fibrosis amount, the rate of reentrant waves significantly decreased and higher levels of regularity and coupling were observed (*p* < 0.0001). Higher spatial variability and pattern stochasticity over repetitions was observed for larger amount of fibrosis, especially in the presence of patchy and compact fibrosis. Overall, propagation slowing and organization led to higher stability of re-entrant waves. These results strengthen the evidence that the amount and spatial distribution of fibrosis concur in dictating re-entry dynamics in remodeled tissue and represent key factors in AF maintenance.

## Introduction

Atrial fibrillation (AF) is the most common sustained arrhythmia in the clinical practice. The current estimated prevalence of AF is between 2 and 4% and is expected to rise by 2.3 fold in 2050, due to population aging^1^. AF is associated with a five-fold increase in the risk of stroke^2^ and often coexists with other pathologies, such as heart failure, causing augmented morbidity and mortality^1^.

AF is a multifactorial disease^3,4^, characterized by a high-rate and disorganized activation of the atria, which determines irregular, often rapid, ventricular activation^5^. This complex atrial activity is related to different mechanisms, which include high-frequency ectopic foci, multiple wavelets, and rotors^3,4^. These mechanisms are promoted by electrophysiological and structural alterations, which contribute to the formation of an arrhythmic substrate^3,4^.

Accumulating experimental and clinical evidence suggests the role of atrial fibrosis in the structural remodeling that favorites AF^6,7^. Fibrosis is a scarring process, characterized by cardiac fibroblast activation and differentiation into myofibroblasts, loss of extracellular matrix homeostasis, and excess deposition of collagen^6,8^. Atrial fibrosis may be the consequence of AF-induced remodeling, of an underlying structural heart disease and/or aging, or of an atrial cardiomyopathy with genetic etiology that precedes the arrhythmia^6,9,10^. Fibrosis-related structural alterations contribute to AF mechanisms by altering impulse formation and propagation^3,6^. Fibrosis alters wave propagation by determining heterogenous and/or slow conduction, unidirectional blocks and barriers to wave propagation, and by providing anchoring points for re-entries^3,6^. Arrhythmogenic effects depend on the overall amount of fibrosis, but also on its texture and spatial distribution^11–13^. Fibrosis is not a single entity and its different forms (i.e., patchy, diffuse, and compact) determine specific conduction abnormalities^8,14^. Patchy/interstitial fibrosis, which is characterized by elongated non-conducting collagen strands, plays a major arrhythmogenic role, being involved in conduction slowing, augmented anisotropy, unidirectional block, and zig-zag conduction between bundles. Diffuse fibrosis, consisting of short non-conducting collagen segments, determines a reduction of conduction speed and leads to the formation of reentrant waves. Compact fibrosis, characterized by large areas completely deprived by myocytes, seems less arrhythmogenic, but it can provide anchoring points for re-entrant waves^8^.

Despite accumulating knowledge, gaps still exist in our understanding of the specific relationship between distinct characteristics and spatial distributions of fibrosis and the resulting arrhythmogenic substrates. Computer models may offer a unique tool to investigate the effects of fibrosis on wave propagation, granting direct control on fibrosis properties and independence from other factors^15–18^. Diverse computational directions have been pursued to investigate different aspects of fibrosis action at different scales and levels of detail. The first modeling direction focuses on the microscopic description of the cellular and tissue mechanisms of fibrosis, implementing detailed models of fibrotic cell types, ion channel modulation, and conduction disturbances^19–21^. The second approach aims to develop accurate patient-specific models to identify the specific AF driver regions in the single patient for patient-oriented therapies^22–32^. Model adaptation to patient-specific data mainly relies on imaging-derived anatomies and fibrosis distributions, which are mostly assumed as transmural conduction blocks, given the limited resolution of clinically-available imaging modalities. Challenges to model personalization rely on precise definition of fibre orientation and atrial thickness profiles^12,25,28,32,33^, as well as on the integration of intramural fibrosis distribution based on either sparse experimental data or simplified assumptions^12,25,34,35^. The third modelling approach aims to identify general, instead of specific, properties of wavefront-fibrosis interactions^36–42^. This is pursued by performing systematic generation of fibrosis patterns under controlled conditions and evaluating the emerging behaviors in a statistical sense. To dissect the effects of fibrosis from other factors, these studies are usually performed in anatomically and structurally-simplified models. Previous investigations in 2D lattice ventricular models analyzed the effects of homogeneous^38^ and heterogenous^37^ distributions of diffuse fibrosis or combinations of fibrosis and adipose tissue^43^ on arrhythmia onset and type. Other studies tested the influence of patchy interstitial fibrosis on plane wave propagation^39^ and on dynamical anchoring and organization of rotors^41^, or classified fibrosis arrhythmogenicity based on the geometrical features of the fibrotic pattern^36^. Recently, the stabilizing effect of local scars or fibrosis regions on rotor meandering was analyzed in the presence of noise in a stochastic mathematical model of AF^42^. To our knowledge, no systematic study has been performed yet, which quantified the effects of multiple geometric features of fibrosis on re-entry dynamics and stability in electrically-remodeled atrial tissues.

To fill this gap, we performed an extensive in-silico study, where we systematically analyzed how changes in fibrosis distribution and type affected the rhythmic, regularity, and stability properties of re-entrant waves in atrial tissue. We implemented a stochastic algorithm that generated fibrosis random patterns with tunable amount, size, and orientation of fibrotic elements, mimicking diffuse, compact, and patchy fibrosis. The generated fibrotic patterns were integrated on a spheric monolayer of atrial tissue with Courtemanche-Ramirez-Nattel (CRN) ionic dynamics^44^. A total of 245 simulations were systematically run at changing atrial fibrosis features, and the properties and stability of the emerging propagation patterns were evaluated. A quantitative description of the patterns, in terms of clinically interpretable features, was obtained applying frequency-domain analysis to synthetic bipolar electrograms (EGMs) extracted from each simulation.

## Methods

**Figure 1:**
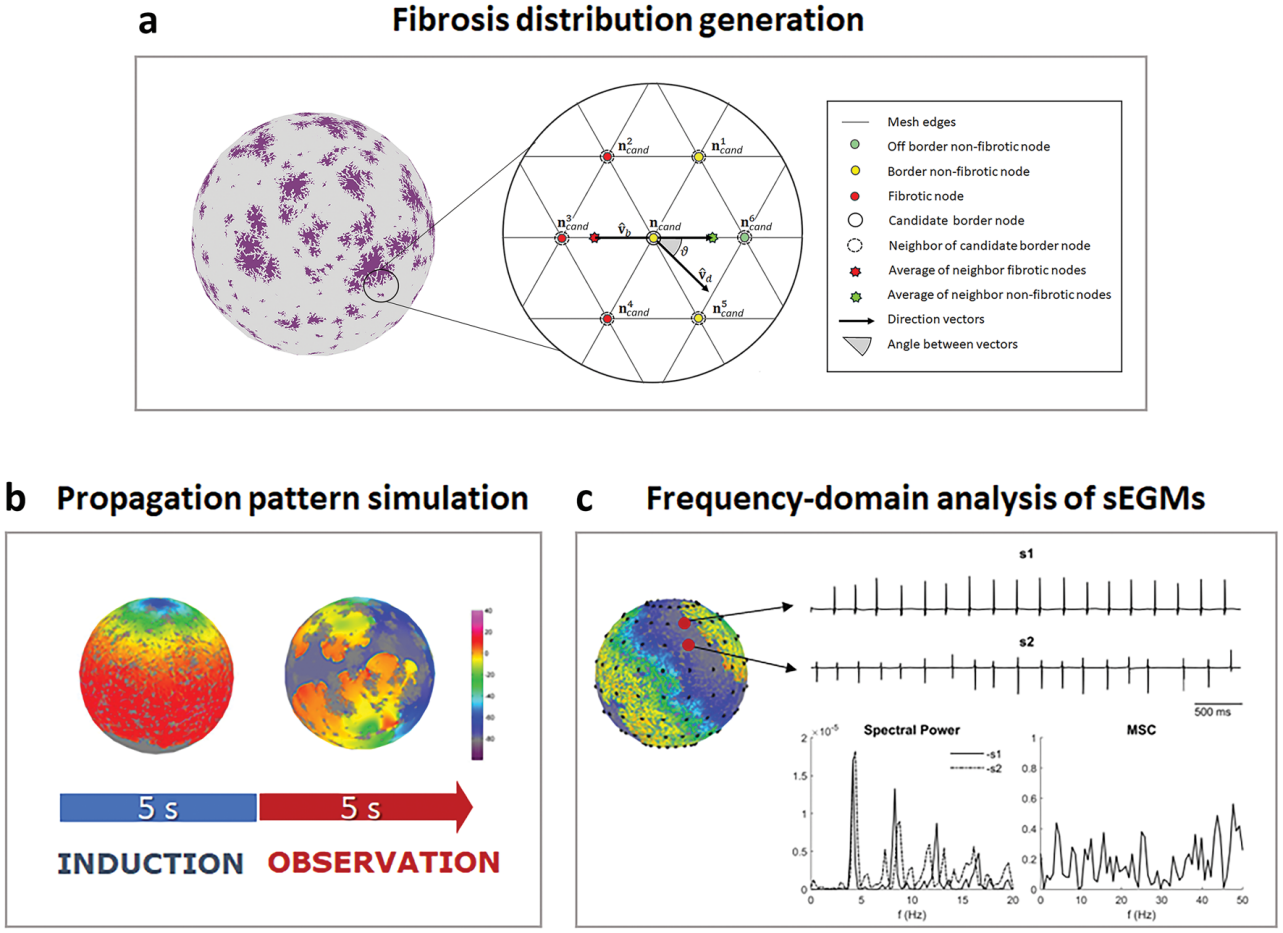
Scheme of the systematic computer simulation approach. (**a**) Stochastic generation of fibrotic patterns. On the left, sphere with fibrotic pattern (in purple). On the right, magnification of the mesh and highlight of the candidate node and neighbor nodes considered in the process of node attribution to fibrotic regions. See text for details. (**b**) Simulation protocol composed by an induction window under high-frequency pacing, and an observation window with propagation pattern free evolution. (**c**) Generation and frequency-domain analysis of synthetic bipolar electrograms (sEGMs: s1, s2) and computation of power spectra and magnitude squared-coherence (MSC) to extract rate, organization, and coupling information.

The study procedure is schematized in Fig. 1 and described in the next sections. Systematic simulations were run on a spherical atrial tissue model (Sect. “Electrophysiological model and tissue geometry” ) and fibrotic spatial patterns were generated by the stochastic algorithm (Fig. 1a, Sect. “Fibrotic model and stochastically-generated patterns” ). Simulations were performed according to a protocol, which comprised an induction window and an observation window (Fig. 1b, Sect. “Simulation protocol” ). During the observation window, pattern stability was assessed, and pattern rate, regularity, and coupling were determined from synthetic EGMs (Fig. 1c, Sect. “Synthetic signal generation and analysis” ). Computational models and analysis tools were implemented in Matlab programming language (The MathWorks, Inc., Natick, MA, USA).

### Electrophysiological model and tissue geometry

The CRN cell model for the human atrial action potential was used to describe cell dynamics^44^. The total ionic transmembrane current $I_{ion}$ is modeled by:

1 $$I_{ion} = I_{Na} + I_{K1} + I_{to} + I_{Kur} + I_{Kr} + I_{Ks} + I_{Ca,L} + I_{p,ca} + I_{NaK} + I_{NaCa} + I_{b,Na} + I_{b,Ca}$$

where $I_{Na}$ is the fast inward ${\text{sodium}}$ current, $I_{K1}$ the inward rectifier ${\text{potassium }}$ current, $I_{to}$ the transient outward potassium current, $I_{Kur}$, $I_{Kr}$, and $I_{Ks}$ are the ultrarapid, rapid and slow delayed rectifier potassium currents, respectively, $I_{Ca,L}$ is the L-type ${\text{calcium}}$ current, $I_{p,ca}$ the calcium pump current, $I_{NaK}$ the sodium–potassium pump current, $I_{NaCa}$ the sodium-calcium exchanger current, and $I_{b,Na}$ and $I_{b,Ca}$ are the ${\text{sodium }}$ and calcium background currents, respectively.

Cell coupling was implemented following a monodomain formulation^45^, leading to the reaction–diffusion ODE-PDE equation:

2 $$\frac{\partial V}{{\partial t}} = \nabla \cdot \left( {D_{T} \nabla V} \right) - \frac{{I_{ion} }}{{C_{m} }} + \frac{{I_{st} }}{{C_{m} }}$$

where $V$ is the transmembrane voltage, $\nabla$ denotes the partial derivative operator, $\cdot$ is the dot product, *I*_*st*_ is the stimulus current flowing across the membrane, *C*_*m*_ is the total membrane capacitance, and ${\mathbf{D}}_{T}$ is the diffusion tensor, given by the ratio of the effective conductivity tensor to the surface-to-volume ratio.

Ionic parameters were adjusted to mimic electrically-remodelled atrial tissue features, as previously suggested^46^. Specifically, the currents *I*_*to*_, *I*_*CaL*_, and *I*_*Kur*_ were reduced by 80%, 30%, and 90%, respectively, and *I*_*Kr*_ was increased by 50%, to reduce action potential duration (APD) and increase the maximal slope of APD restitution curve. Diffusion was set uniform and isotropic with D_T_ = 1·10^–3^ cm^2^/ms. The model integration domain was a monolayer sphere of 10 cm diameter, discretized into a triangular mesh comprising ~ 290 K nodes with spatial resolution of about 280 μm.

### Fibrotic model and stochastically-generated patterns

Similar to other works^36,36–38^, fibrotic elements were integrated in the tissue model by replacing mesh nodes with non-excitable obstacles with no-flux boundary conditions. A previously-proposed stochastic algorithm^47^ was extended to generate random fibrosis patterns with tunable amount, size, and orientation of fibrotic elements. Increasing element size reproduced transitions from diffuse to compact fibrosis, while increasing element orientation mimicked the appearance of patchy/interstitial fibrosis. The stochastic generation was based on an iterative procedure, controlled by three parameters: the overall amount or density of fibrosis $D$, expressed as percentage of blocked nodes, the probability parameter *p*_*th*_, which tuned the size of fibrotic elements, and the anisotropy level *α*, which tuned fibrotic element orientation according to a predefined direction.

Given the set **n**_*k*_ of the position vectors of the *k* = 1…$N$ nodes of the mesh, and the class function *c* assigning each node to the values 0 or 1, based on its conductive properties:

3 $$c\left( {{\varvec{n}}_{k} } \right) = \left\{ {\begin{array}{*{20}c} 0 & {if\; k \;is \;a \;conductive\; node} \\ 1 & {if\; k\; is\; a\; fibrotic \;node } \\ \end{array} } \right.$$

at each step of the procedure the temporary fibrosis density was computed as:

4 $$D_{temp} = 100\frac{{\mathop \sum \nolimits_{k = 1}^{N} c\left( {{\mathbf{n}}_{k} } \right)}}{N}$$

Until the condition $D_{temp} < D$ was fulfilled, a new candidate node **n**_cand_ was drawn from the set of non-fibrotic nodes (i.e., **n**_i_ with c(**n**_i_) = 0), with constraints given by two stochastic conditions.

The first condition determined the selection basin of **n**_cand_ and was tuned by the probability threshold 0 ≤ *p*_*th*_ ≤ 1, according to:

5 $$\left\{ {\begin{array}{*{20}c} {q > p_{th} } & { \to {\mathbf{n}}_{cand} { } \;drawn \;from \;I_{border}^{{}} } \\ {q \le p_{th } } & { \to {\mathbf{n}}_{cand} { } \;drawn \;from\; I_{border}^{C} } \\ \end{array} } \right.$$

where *q* is a random number drawn from the uniform distribution *U* [0 1], $I_{border}$ is the list of the indices of the nodes that belong to the external border of fibrotic elements (i.e., conductive nodes with at least one fibrotic neighbor node) and $I_{border}^{C} \;{\text{is}}\;{\text{ the}}\;{\text{ set }}\;{\text{of }}\;{\text{conductive}}\;{\text{ nodes }}\;{\text{complementary}}\;{\text{ to}}\;{ }I_{border}$. According to Eq. 5, a new node was added to the border of a pre-existing fibrotic element only when *q* was larger than *p*_*th*_, while a new fibrotic element was created otherwise. Thus, the choice of small *p*_*th*_ values allowed the formation of larger fibrotic elements, while large *p*_*th*_ values resulted in smaller fibrotic elements. To ensure that the fibrotic elements were simply connected (i.e., they did not present holes) and to avoid the blending of distinct fibrotic regions, a selected border node was discarded if its fibrotic neighboring nodes did not form a connected set.

The second condition controlled the position of border candidate nodes and was tuned by the anisotropy threshold α ≥ 0:

6 $$r < \left| {{\hat{\mathbf{v}}}_{d} \cdot {\hat{\mathbf{v}}}_{b} } \right|^{\alpha }$$

where $r$ is a random number extracted from the uniform distribution *U* [0 1], · indicates the dot product, ${\hat{\mathbf{v}}}_{d}$ is the versor, parallel to the mesh, that indicates the predefined orientation of fibrosis, and ${\hat{\mathbf{v}}}_{b}$ is the versor of the border normal at the position ${\mathbf{n}}_{cand}$. ${\mathbf{v}}_{b}$ was operatively determined through the equations:

7a $${\mathbf{v}}_{b} = {\mathbf{v}}_{{\varvec{b}}}^{\user2{^{\prime}}} - {\mathbf{v}}_{{\varvec{b}}}^{\user2{^{\prime}}} \cdot {\hat{\mathbf{s}}}_{{}}$$

7b $${\mathbf{v}}_{{\varvec{b}}}^{\user2{^{\prime}}} = \frac{{\mathop \sum \nolimits_{j = 1}^{M} \left( {1 - c\left( {{\mathbf{n}}_{cand}^{j} } \right)} \right){\mathbf{n}}_{cand}^{j} }}{{\mathop \sum \nolimits_{j = 1}^{M} \left( {1 - c\left( {{\mathbf{n}}_{cand}^{j} } \right)} \right)}} - \frac{{\mathop \sum \nolimits_{j = 1}^{M} c\left( {{\mathbf{n}}_{cand}^{j} } \right){\mathbf{n}}_{cand}^{j} }}{{\mathop \sum \nolimits_{j = 1}^{M} c\left( {{\mathbf{n}}_{cand}^{j} } \right)}}$$

where ${\mathbf{n}}_{cand}^{j}$ are the position vectors of the $j = 1 \ldots M$ neighboring nodes of ${\mathbf{n}}_{cand}$, $c$ is the class function defined above, and ${\hat{\mathbf{s}}}_{{}}$ is the versor of the surface normal calculated at ${\mathbf{n}}_{cand}$. As schematized in Fig. 1a, ${\mathbf{v}}_{{\varvec{b}}}^{\user2{^{\prime}}}$ was calculated as the difference between the average position (green star) of the non-fibrotic neighbors of ${\mathbf{n}}_{cand}$ (encircled green and yellow dots) and the average position (red star) of the fibrotic neighbors of **n**_cand_ (encircled red dots). The border normal vector ${\mathbf{v}}_{b}$ was thus calculated by orthogonalizing ${\mathbf{v}}_{{\varvec{b}}}^{\user2{^{\prime}}}$ to the surface normal ${\hat{\mathbf{s}}}_{{}}$.

The stochastic condition in Eq. 6 is always fulfilled for α = 0, allowing fibrotic elements to grow in random directions. At increasing α, Eq. 6 instead favors fibrosis growth along directions aligned to or forming small angles θ with ${\widehat{\mathbf{v}}}_{d}$ (see Fig. 1a), leading to the formation of oriented, stringy elements. The algorithm computational efficiency was optimized by precomputing node connectivity and surface normals and by updating the border list $I_{border}$ only after a certain fraction of its nodes ($f_{update}$ = 5%) was added to the fibrotic element. The pseudocode of the algorithm is reported in the Table S1 in the Supplementary Material.

The stochastic generation algorithm was tested to assess its reliability in generating fibrotic patterns with tunable properties at the change of the input parameters (*D, p*_*th,*_, α). The control exerted by the three parameters was analyzed in terms of the generated output density, the dimension of the generated fibrosis patches, and the *θ*-angle distribution of accepted nodes as a surrogate measure of pattern anisotropy. Validation details are presented in the Supplementary Material.

For the simulations, a set of fibrosis spatial distributions was generated by changing the three input parameters in predefined ranges. The density *D* was changed over the values 3, 5, 12, 21, 30 and 40%, to cover fibrosis-based clinical patient classes (i.e., Utah stages)^48^. The size parameter p_th_ was varied over the empirically-chosen values 0.008, 0.01, 0.015, 0.03, 0.05, 0.07, 0.1, to obtain fibrotic elements with progressively smaller average size and lower number of nodes (see Sect. “Fibrosis pattern generation” and Supplementary Material). Simulations were run for all the combinations of *D* and *p*_*th*_ (42 combinations) setting *α* = 0, i.e., producing diffuse to compact fibrosis distributions. Based on previous results^39^, the effect of patchy/interstitial fibrosis was evaluated only for large fibrosis amounts (*D* = 30%) at all element size (7 combinations) by empirically set *α* = 4 (configuration indicated by 30or). For each of the considered 49 combinations, the stochastic generation was repeated 5 times, leading to a total of 245 fibrotic models, which were used in the simulations.

### Simulation protocol

The same simulation protocol was applied in all the 245 fibrosis models (Fig. 1b). The protocol was composed of an induction window and an observation window, both of 5 s duration. During the induction window, propagation patterns were started and maintained by a high-frequency stimulation source at the sphere pole. Like in previous studies^19,36^, the stimulation frequency was adapted so that a new pulse was released as soon as the tissue was re-excitable. During the observation window, stimulation was switched off and propagation patterns evolved freely. The stability and characteristics of wave propagation patterns were assessed in the observation window. A pattern was considered stable if excitation persisted through the whole observation window. The rate, regularity, and coupling properties of stable patterns were quantified by generating and analyzing synthetic electrograms (Sect. “Synthetic signal generation and analysis” ).

### Numerical integration

ODE-PDE system integration was performed by a previously-validated, fully-adaptive multi-resolution algorithm^49^, which dynamically restricted the computation to a set of active nodes reducing computational burden while preserving simulation accuracy. Reaction and diffusion were integrated with time step Δt = 0.1 ms, using the Rush Larsen non-standard finite difference forward Euler method and explicit node-centered finite difference stencils^50^, respectively.

### Synthetic signal generation and analysis

Simulated bipolar signals were generated during the observation window of each simulation and analyzed in the frequency-domain to provide a quantitative description of propagation pattern properties. A detailed description of the generation and analysis of the signal is provided in the Supplementary Material.

Briefly, in each simulation, a set of 144 synthetic bipolar EGMs of 5 s length (sampling frequency of 1 kHz) was generated, using the current source approximation^46^, from a grid of virtual electrodes, composed by eighteen regularly spaced splines, each carrying eight regularly spaced bipoles (Fig. 1c). The first 500 ms were discarded to let the patterns stabilize and the remaining signal segments were analyzed. The peak-to-peak (P2P) voltage amplitude of each EGM was extracted and used as a correlate of fibrosis presence^51^.

Frequency-domain analysis was applied to extract the rhythmic properties of the EGMs, in terms of rate, regularity, and coupling metrics. Rate and regularity metrics were quantified in each EGM in terms of the dominant frequency (DF) and regularity index (RI), as previously proposed^52,53^. DF is defined as the frequency of the maximum of the signal spectral amplitude, and can be assumed as the rate of the activation process. RI is a measure of spectral dispersion and can be assumed as an index of electrogram organization. RI ranged between 0 and 1, with higher values indicating more organized electrical activity. The level of coupling of the electrical activity between neighboring bipoles (on the same spline, or between adjacent splines) was quantified by calculating the coupling index Cxy between pairs of EGMs, which was defined as the mean value of magnitude-squared coherence of the EGMs over the frequency range^54^. Cxy ranges between 0 and 1, with 0 indicating the absence of coupling and 1 maximal coupling.

### Statistical analysis

In each simulation, the average and spatial variability values of P2P and frequency-domain indices were calculated as mean and standard deviation (std) values on all the bipoles or bipole pairs (for Cxy). An average coupling asymmetry index (DCxy) was calculated as the ratio between the mean line-wise (i.e., on sphere parallels) and spline-wise (i.e., on sphere meridians) Cxy values, and its variability was computed by error propagation formulas. DCxy values higher than one indicated higher coupling between neighboring splines, while values lower than one indicated higher coupling along splines.

The association between EGM-based properties (average and variability values of P2P, DF, RI, Cxy, and DCxy) or pattern stability and the geometrical features of fibrosis was assessed using generalized linear models. The effects of fibrosis amount and size were evaluated on the simulation subset with unoriented fibrotic elements (*α* = 0), while those of orientation were assessed on the simulation subset with *D* = 30%, in the absence (*α* = 0) and presence of patchy/stringy fibrosis (α = 4). Details on the calculation of the models are reported in the Supplementary Material. Post-hoc comparisons were performed in the presence of statistically-significantly associations. P-values for model factors and in post-hoc comparisons were corrected for multiple comparisons using Bonferroni correction. A p-value < 0.05 was considered statistically significant.

## Results

### Fibrosis pattern generation

**Figure 2:**
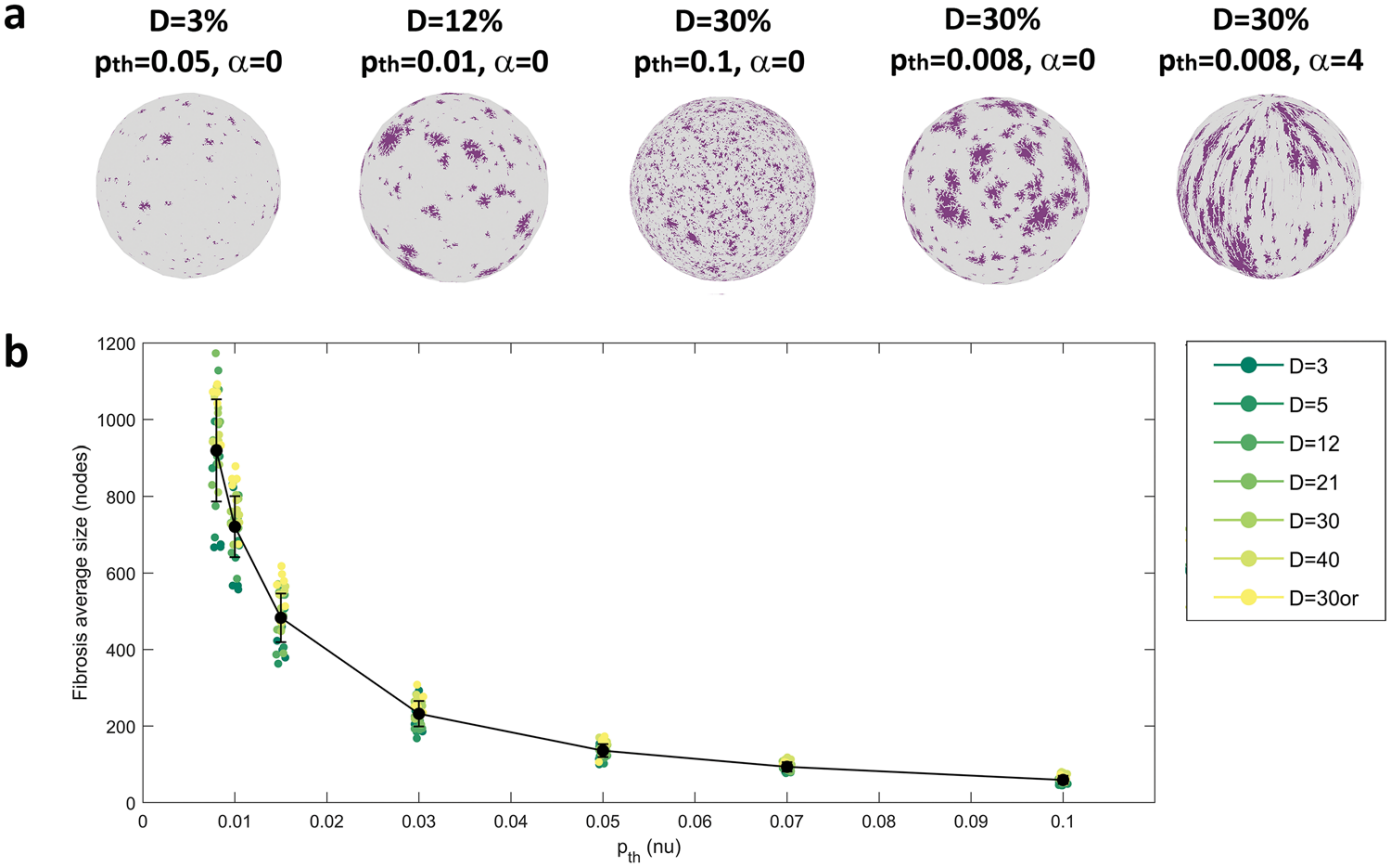
Generation of fibrosis patterns. (**a**) Exemplary fibrotic patterns (in purple) obtained setting the stochastic algorithm parameters *D*, *p*_*th*_, and *α* to the indicated values. From left to right: very-low density fibrosis, low-medium density fibrosis, high density diffuse fibrosis, high density compact fibrosis, high density patchy fibrosis. (**b**) Distribution of the weighted mean size (expressed in terms of mesh nodes) of fibrosis elements from the stochastic realizations used in the simulation, displayed as a function of the size parameter *p*_*th*_, for different fibrosis densities (indicated in different colors). Black dots and whiskers indicate average values and standard deviation over simulation subgroups, while small colored dots correspond to single simulation outcomes.

Figure 2a shows examples of fibrosis patterns generated for different tuning of the stochastic algorithm parameters. The total amount of fibrosis (indicated in purple) progressively increased from left to right, passing from *D* = 3% to 30%. For the same amount of fibrosis, diffuse fibrosis was obtained at larger *p*_*th*_ values (*p*_*th*_ = 0.1, center), while larger fibrotic regions were obtained for small values of *p*_*th*_ (*p*_*th*_ = 0.008, right panels). Compact fibrotic elements were obtained for null α values, while patchy fibrotic elements for *α* = 4 (rightmost panel). The quantitative evaluation of the stochastic algorithm capability to generate different fibrosis patterns at changing input parameters is presented in the Supplementary Material, while the weighted mean size of the generated pattern elements used in the simulations is shown in Fig. 2b. The validation results showed that the stochastic algorithm was able to generate fibrosis patterns of predefined density *D*, with limited average bias (Fig. S1a). The increase of the size parameter *p*_*th*_ determined a progressive increase in the number of the generated fibrotic elements (Fig. S1b) and decrease in the average (Fig. S1c and Fig. 2b) and maximal dimension (Fig. S1d) of the elements and in their size dispersion (Fig. S1e). Dimensions increased at increasing *D*, with larger variations at small *p*_*th*_ values and more marked effects for maximal size and size dispersion. The increase of the anisotropy parameter α from 0 to 4 determined a marked restriction of the accepted angles θ for border nodes, with a shift from a uniform angle distribution to a zero-peaked narrow distribution, consistent with the selection of the preferential orientation.

### Propagation patterns emerging at different fibrosis distributions

**Figure 3:**
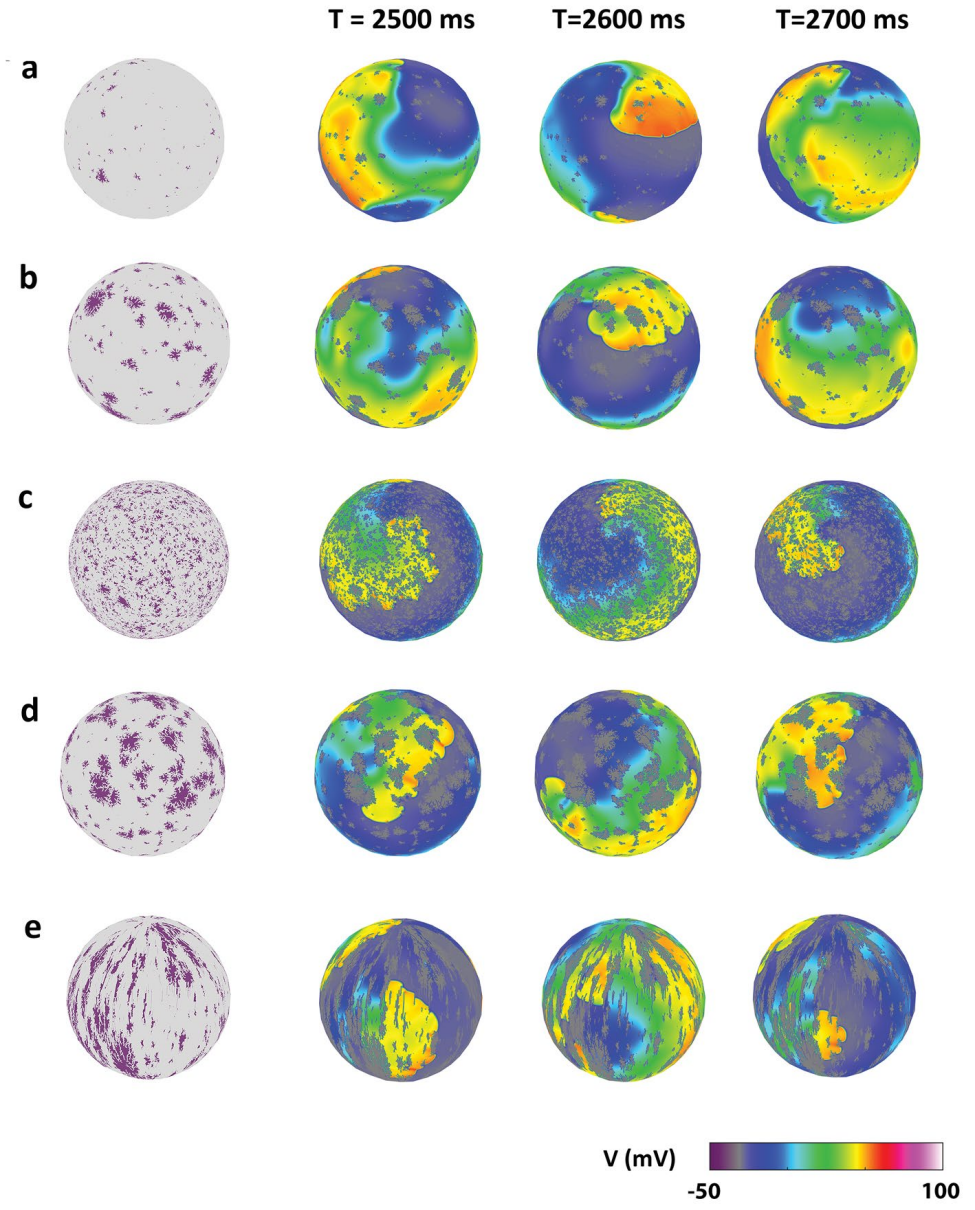
Exemplary propagation patterns emerging for different geometrical features of fibrosis. Subsequent snapshots of action potential maps are indicated in color code. Spiral wave meandering and break-up in the presence of very-low (**a**) and medium–low densities of fibrosis (**b**). Stable spiral wave without break-up at high-density diffuse fibrosis (**c**). Spiral wave fragmentation in the presence of a high-density of compact fibrosis (**d**). Wave canalization in the presence of a high-density of patchy fibrosis (**e**).

**Figure 4:**
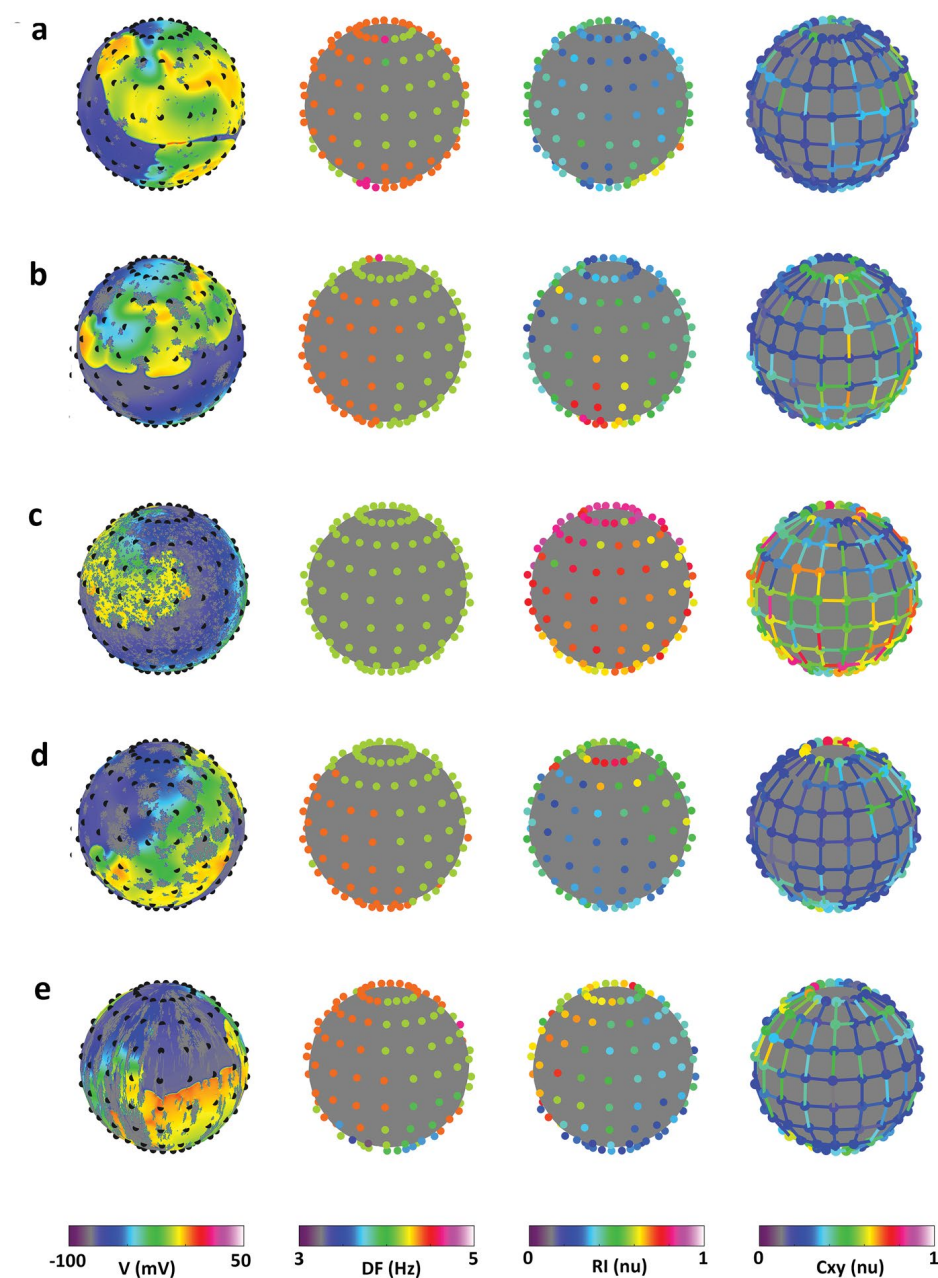
Quantification of propagation patterns properties in the frequency-domain. For each propagation pattern (rows, corresponding to patterns in Fig. 3), a snapshot of the activation pattern is shown together with dominant frequency (DF), regularity index (RI), and coupling (Cxy) maps (displayed from left to right). Meandering spiral waves (**a, b**) present the highest rate, and the lowest regularity and coupling. Stable spiral waves (**c**) present the slowest rate and the highest regularity and coupling. Wave fragmentation (**d**) is associated with an intermediate rate, and low regularity and coupling. Wave canalization is characterized by intermediate rate and low regularity, with higher coupling along splines than lines.

An overview of the variety of propagation patterns observed at changing fibrosis features is presented through the exemplary patterns in Fig. 3. In the presence of small amounts of fibrosis (*D* = 5 and 12% in Fig. 3a, b, respectively), propagation was characterized by meandering spirals, which gave rise to break-up and split into multiple waves. The increase of fibrosis amount (*D* = 30% in Fig. 4c–e) led to an overall slowing of the propagating waves, albeit with the emergence of various propagation patterns for different fibrosis textures. Slow and very regular re-entrant waves, without meandering nor break-up, are shown in Fig. 4c in the presence of uniformly distributed diffuse fibrosis. The fragmentation of spiral waves by larger-size compact fibrosis is displayed in Fig. 4d. Anisotropic wave canalization is observable in Fig. 4e, where stringy fibrosis patches favored propagation along and hinder propagation across fibrosis direction.

### Electrogram-based quantification of propagation pattern properties

The frequency-domain analysis of EGMs for the described patterns is displayed in Fig. 4 in terms of DF, RI, and Cxy maps. At low fibrosis densities (a and b), EGMs displayed high voltage amplitudes (P2P = 0.167 ± 0.034 au and 0.156 ± 0.049 au for a and b, respectively). Spiral break-up patterns were characterized by high activation rates (DF = 4.31 ± 0.14 and 4.23 ± 0.12 Hz for a and b, respectively), and low regularity (RI = 0.40 ± 0.10 nu and 0.44 ± 0.12 nu for a and b, respectively) and coupling (Cxy = 0.27 ± 0.09 nu and 0.34 ± 0.13 nu for a and b, respectively). At higher fibrosis densities, EGM displayed lower voltage amplitudes (P2P = 0.097 ± 0.044 au, 0.121 ± 0.064 au, and 0.116 ± 0.066 au, for c, d, and e, respectively). The single, regular, spiral wave (c) displayed the lowest activation rate (DF = 4.15 Hz) and the highest regularity (RI = 0.74 ± 0.09 nu) and coupling (Cxy = 0.54 ± 0.17 nu). Wave fragmentation (d) led to slightly higher activation rate (DF = 4.23 ± 0.12 Hz) and lower regularity (RI = 0.48 ± 0.13 nu) and coupling (Cxy = 0.37 ± 0.16 nu). Wave canalization (e) showed indices (DF = 4.27 ± 0.24 Hz, RI = 0.52 ± 0.16 nu, Cxy = 0.37 ± 0.15 nu) similar to wave fragmentation, but coupling was higher along splines than lines, resulting in an asymmetry index of 0.89 au (versus 1.13 au in panel d).

**Figure 5:**
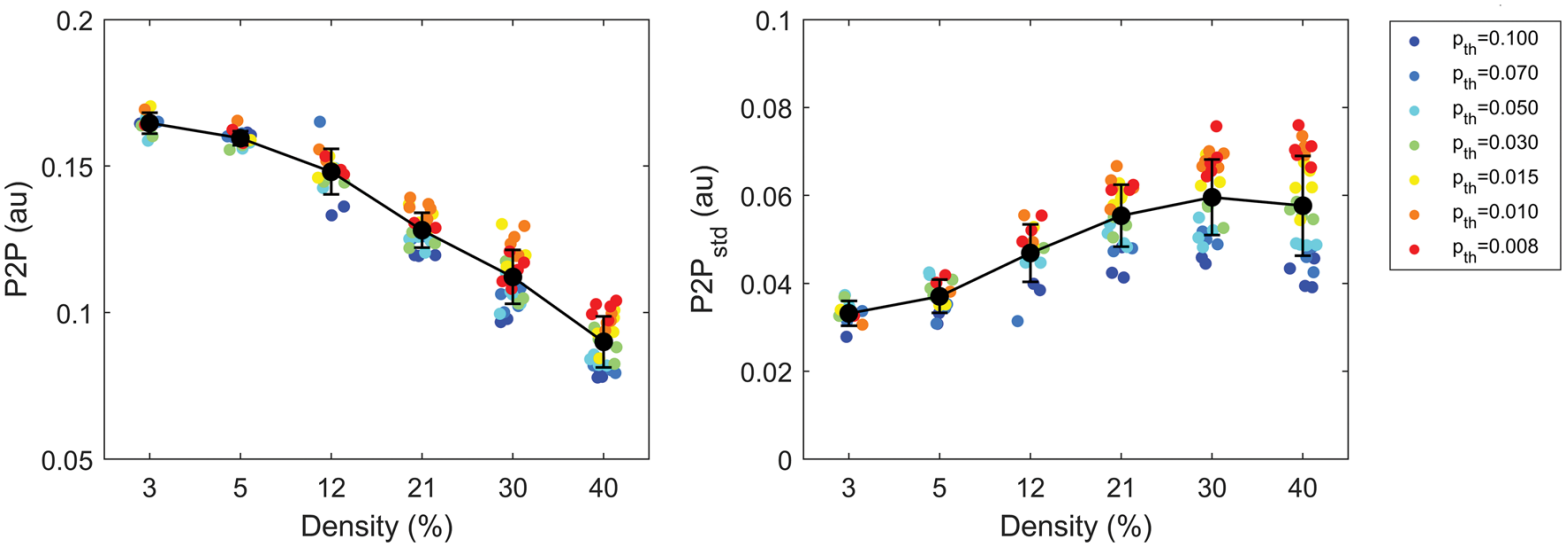
Voltage amplitude of synthetic electrograms for different fibrosis amounts and sizes. Average values (P2P, left panel) and spatial variability values (P2P_std_, right panel) of electrogram voltage amplitude are displayed as a function of fibrosis overall amount (density, *D*) and color-coded according to fibrosis size parameter (*p*_*th*_). Black dots and whiskers indicate mean values and standard deviations over simulation subgroups, while small colored dots correspond to single simulation outcomes. Only simulations obtained for diffuse and compact fibrosis (*α* = 0) are shown. The increase of fibrosis density determined a decrease in voltage amplitude and increase in voltage amplitude spatial variability.

**Figure 6:**
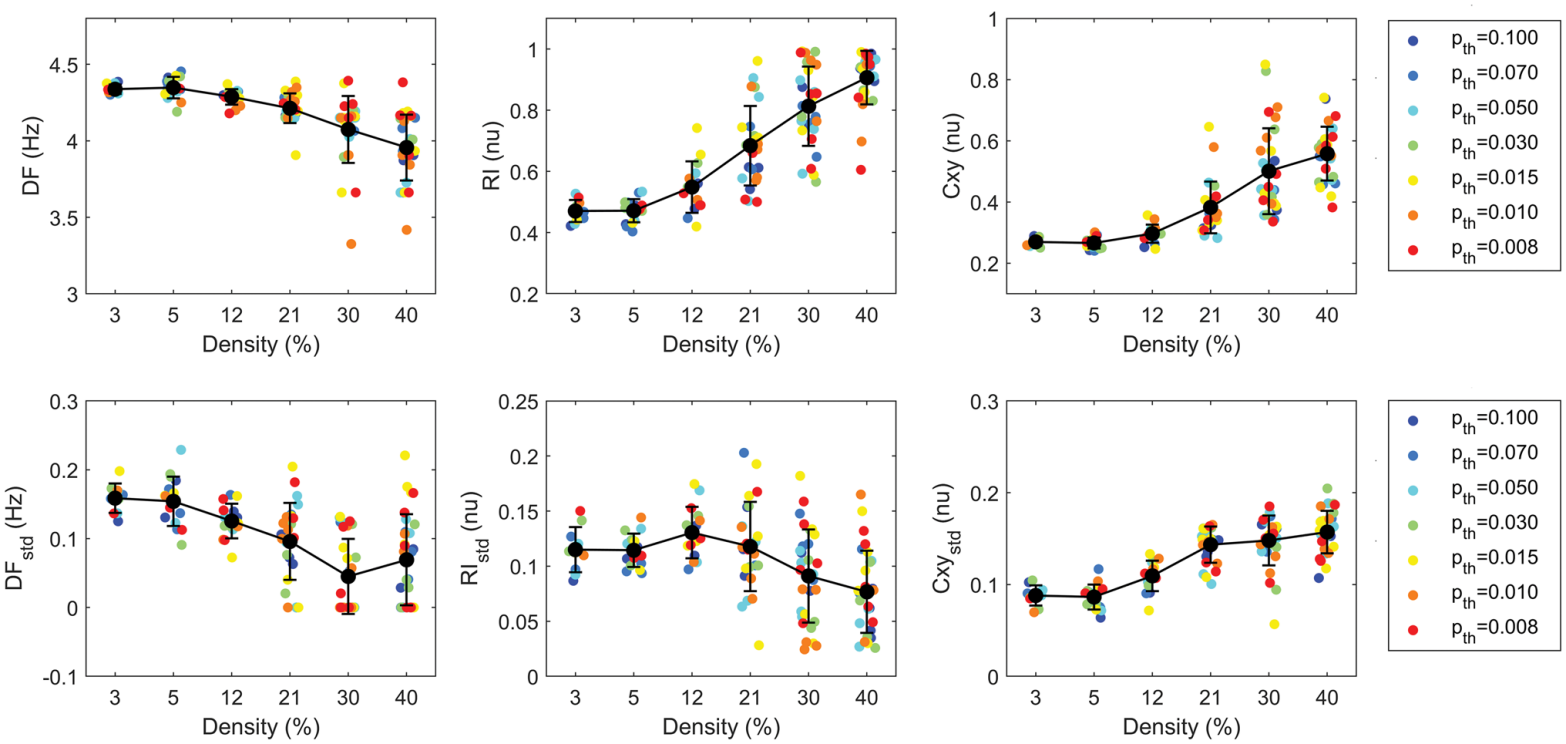
Frequency-domain properties of synthetic electrograms for different fibrosis amounts and sizes. Average values (top panels) and spatial variability values (std, bottom panels) of rate (DF and DF_std_), regularity (RI and RI_std_) and coupling (Cxy and Cxy_std_) are shown as a function of fibrosis overall amount (density, D) and color-coded according to fibrosis size parameter (*p*_*th*_). Black dots and whiskers indicate mean values and standard deviations over simulation subgroups, while small colored dots correspond to single simulation outcomes. Only simulations obtained for diffuse and compact fibrosis (α = 0) are considered. The increase of fibrosis amount determines slowing and organization of wave propagation, with an overall decrease of DF and DF_std_ and increase in RI, Cxy, and Cxy_std_, albeit with an increased stochasticity of simulation outcomes.

The overall results of EGM-based analysis for diffuse and compact fibrosis textures (α = 0) are summarized in Figs. 5 and 6 and Tables S2 and S3. The mean voltage amplitude and its spatial variability showed a statistically significant (*p* < 0.00001) dependence on fibrosis amount *D* (Table S3). The increase of fibrosis from *D* = 3% to 40% led to a progressive decrease of the mean voltage amplitude from P2P = 0.165 ± 0.004 to 0.090 ± 0.009 au (Fig. 5, left) and increase in voltage spatial variability from P2P_std_ = 0.033 ± 0.003 to 0.058 ± 0.011 au (Fig. 5, right). Statistically-significant differences in P2P and P2P_std_ (Table S3) were observed among all fibrosis levels (*p* < 0.001), except for *D* = 3% versus 5% (for P2P and P2P_std_) and *D* = 40% versus 21% and 30% (for P2P_std_). A secondary significant (*p* < 0.00001) modulation of P2P and P2P_std_ values by size parameter *p*_*th*_ was observed, where larger mean and variability values occurred for larger fibrotic obstacles. Statistically-significant differences were mainly observed between larger fibrosis sizes (*p*_*th*_ ≤ 0.015) versus medium-size-to-diffuse (*p*_*th*_ ≥ 0.03). All frequency-domain indices displayed a significant dependence (*p* < 0.00001) on fibrosis amount *D*. A progressive slowing and organization of the propagation patterns was observed at increasing *D* values (Fig. 6). When fibrosis increased from *D* = 3% to 40%, activation rate progressively decreased from DF = 4.34 ± 0.03 to 3.96 ± 0.22 Hz, regularity increased from RI = 0.47 ± 0.04 to 0.91 ± 0.09 nu, coupling increased from Cxy = 0.27 ± 0.01 to 0.56 ± 0.09 nu. The asymmetry index DCxy displayed mean values larger than 1 at all density values, indicating prevalent coupling along lines than splines (Table S2). In pairwise comparisons (Table S3), DF values were significantly higher in the very-low to medium–high fibrosis range (*D* ≤ 21%) than in the high-density fibrosis range (*D* ≥ 30%, *p* < 0.01), and at *D* = 30% than 40% (*p* < 0.05). RI and Cxy were significantly lower (*p* < 0.05) in the low-to-medium fibrosis range (*D* ≤ 12%) than medium–high to high range (*D* ≥ 21%), except for Cxy between *D* = 12% and 21% (*p* = 0.051). Within the *D* ≥ 21% range, RI displayed statistically-significant differences among the three densities (*p* < 0.001), while Cxy only between *D* = 21% and 30% and 40% (*p* < 0.001). The spatial variability of the indices is displayed in the lower panels of Fig. 6. DF_std_ decreased from a maximum value of 0.16 ± 0.02 at *D* = 3% to a minimum value of 0.05 ± 0.06 Hz at *D* = 30%, with statistically-significant differences between medium–high densities (*D* ≥ 21%) versus low densities (*D* ≤ 5%). RI_std_ reached a maximum of 0.13 ± 0.02 nu at *D* = 12% and a minimum of 0.08 ± 0.04 nu at *D* = 40%. Cxy_std_ progressively increased from 0.09 ± 0.01 nu at *D* = 3% to 0.16 ± 0.02 nu at *D* = 40%, with statistically-significant differences between low-to-medium levels (*D* ≤ 12%) and medium–high-to-high densities (*D* ≥ 21%). No statistically-significant association with p_th_ was detected for any frequency-domain index (Table S3 and Figure S3). However, especially for higher densities, index values displayed a larger spread, indicating a higher stochasticity in the simulation outcomes. As shown in Figure S4, variability effects were particularly evident for compact fibrosis (i.e., for lower values of *p*_*th*_ corresponding to larger fibrotic element size) than diffuse fibrosis.

**Figure 7:**
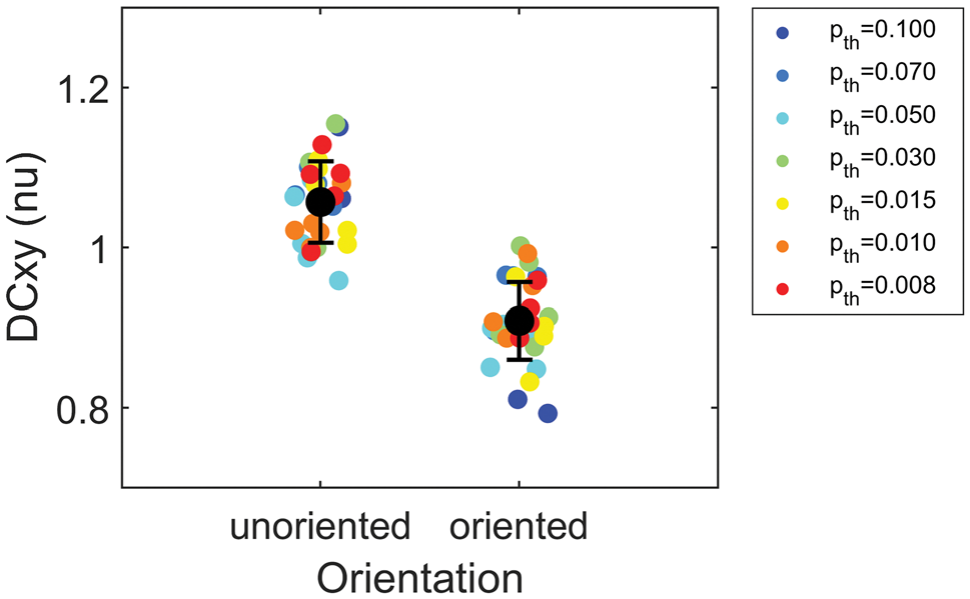
Effects of fibrosis texture on coupling asymmetry of synthetic electrograms. Coupling asymmetry index (DCxy), quantified as the ratio between line-wise and spline-wise coupling values, is compared in diffuse/compact (unoriented) versus stringy/patchy (oriented) fibrotic elements for *D* = 30% fibrosis density. Black dots and whiskers indicate mean values and standard deviations over the simulation subgroups, while small dots correspond to single simulation outcomes and are color-coded according to fibrosis size parameter (*p*_*th*_). Oriented fibrosis determined a prevalent coupling of electrical activity along splines than lines, resulting in asymmetry values lower than one with respect to compact fibrosis where asymmetry values were higher than one.

The effects of patchy fibrosis texture on the patterns are analyzed in Fig. 7 and Table S4, where pattern properties obtained at a density of *D* = 30% were compared for unoriented versus oriented fibrosis textures. No statistically-significant differences were observed between the two conditions in terms of frequency-domain indices. The coupling asymmetry index DCxy showed a statistically-significant association with α (*p* < 0.0001). In patchy fibrosis, DCxy showed values lower than one (DCxy = 0.91 ± 0.05 au), indicating higher coupling along splines than lines, while in diffuse and compact fibrosis asymmetry index values were higher than one (DCxy = 1.06 ± 0.05 au), showing the opposite behavior.

### Stability of propagation patterns

**Figure 8:**
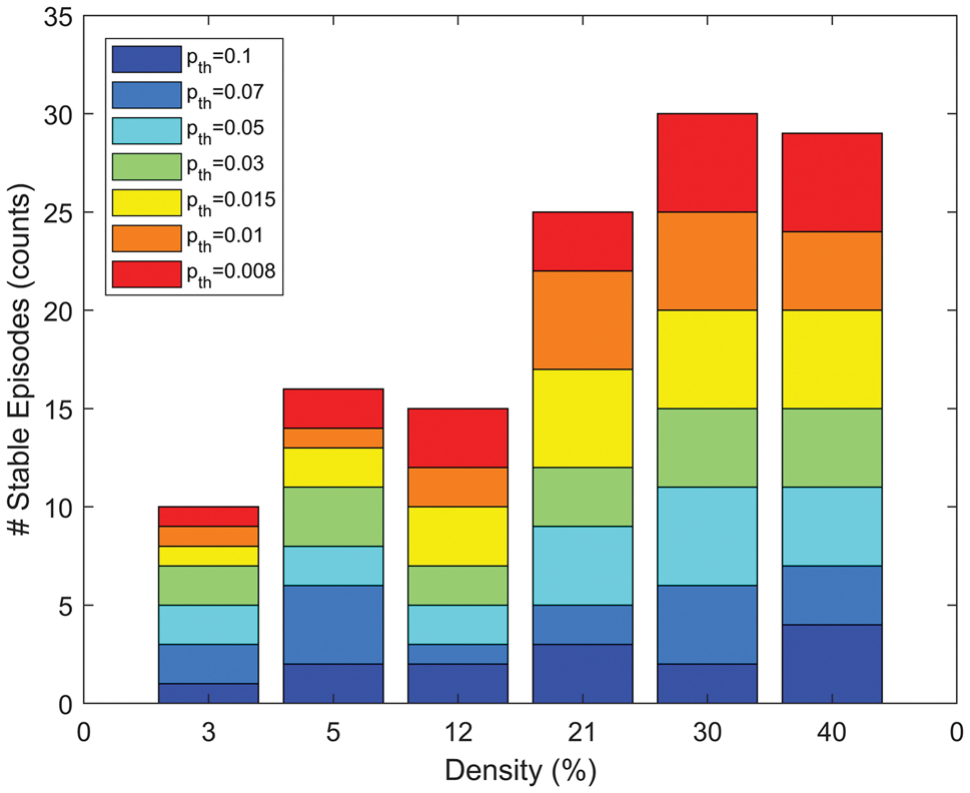
Stability of propagation patterns for different fibrosis amounts and sizes. The number of episodes persisting over the whole observation window is indicated as a function of fibrosis amount (density, *D*) and for different fibrosis sizes (*p*_*th*_ parameter, in color). Only simulations obtained for diffuse and compact fibrosis (*α* = 0) are considered. Fibrosis had a stabilizing effect on propagation patterns, with the number of stable patterns increasing at the increase of fibrosis density.

Stable wave propagation patterns perpetuating along the whole observation window were observed in 159 of 245 simulations (64.9%). Pattern stability was significantly associated with fibrosis overall amount (*p* < 0.0001, Table S3). As shown in Fig. 8, the fraction of stable patterns increased at the increase of fibrosis amount (from a minimum of 28.6% at *D* = 3% to values larger than 70% for *D* ≥ 21%). Statistically-significant differences were observed between the very-low density (*D* = 3%) and medium–high to high fibrosis range (*D* ≥ 21%, *p* < 0.01), between low density (*D* = 5%) and high density (*D* ≥ 30%, *p* < 0.05) and between *D* = 12% and *D* = 30% (*p* < 0.01). Pattern stability displayed no significant dependence on fibrotic element size (Table S3) nor orientation (Table S4).

## Discussion

In this study we performed an extensive evaluation of the effects of different geometrical features of fibrosis on the rhythmic and stability properties of re-entrant waves in an electrically-remodeled atrial tissue. We showed that the progressive increase of fibrosis amount determined: i) a general decrease of the rate and increase of the regularity and coupling of re-entrant waves, although with an increased variety of propagation pattern types and higher dispersion of the indices; ii). an increase in the stability of the patterns.

The aim of our study was focused on the clarification of the sole effects of the fibrosis geometrical features. Thus, like in previous studies^36–39,43^, to better separate the pure effects of fibrosis, we implemented the cell model on a simplified geometry without specific anatomical or structural information, except for fibrosis. We performed our experiments in an extensive and comparative way, progressively varying and comparing the results obtained at different degrees of the property of interest (fibrosis density and patch dimension) or contrasting the effects of a feature (anisotropic fibrotic elements) with the corresponding null model (isotropic elements). The procedure led us to identify general properties of fibrosis-wavefront interactions and appreciate transitions between propagation pattern regimes, which provide meaningful indications, when fibrosis effects can be considered the dominant factor. Thus, our approach differed from patient-specific modelling, where anatomic and structural features are instead essential to predict the exact location of drivers in a specific patient. The generation of fibrosis distribution was inspired by a previously-proposed stochastic pattern generator^47^, that was extended to include the control of fibrosis orientation in addition to amount and size. By tuning of three parameters, our algorithm controlled the amounts of fibrosis and the transitions between fibrosis textures, from diffuse to compact by changing element size, and to patchy by imposing element orientation. Differently from previous approaches^37^, the algorithm did not directly control fibrosis spatial heterogeneity, although, at higher densities of fibrosis, higher homogeneity levels were obtained for diffuse fibrosis than for compact fibrosis. The symmetry of our spheric model was consistent with the random generation of fibrosis elements, since the locations of fibrotic patches were equivalent, and the generated distributions of fibrosis could be considered statistically-equivalent when fixing the parameter set. As in previous studies^36,37^, fibrotic elements were implemented as nodes with zero-flux conditions, mimicking fibrosis macroscopic effect as conduction blocks. Since the adoption of different fibrosis modeling strategies determine effects of different entity on propagation patterns^19,20^, in future studies it would be important to adapt our stochastic algorithm and tissue model to integrate other fibrosis effects. Atrial cell currents were models according to an electrically-remodeled CNR model^46^, where the steep restitution curve produced spontaneous wavefront break-up in the absence of fibrosis. Electrical remodeling precedes and/or accompanies structural remodeling under many disease conditions, thus the patterns observed in this study, arising from the combined effects of electrical and structural remodeling^3^, should better represent reentry dynamics in AF than simply implementing structural alterations. Frequency-domain analysis was applied to synthetic EGMs to provide a quantitative description of the emerging patterns, in terms of clinically-interpretable indices^52,54^. Since DF and RI indices describe solely the rhythmic properties of the activation process, they may be profitably complemented by techniques assessing wave morphology and fragmentation^51,55,56^. As well, information-based measurements may improve coupling estimation allowing propagation tracking through the assessment of causal links between sites^57,58^.

Our results showed a prevalent dependence of propagation pattern characteristics on fibrosis amount, where the increase of fibrosis density determined a reduction in activation rate and overall increase in the regularity and coupling of re-entrant waves, albeit with higher stochasticity of behaviors. The decrease of conduction velocity and DF with fibrosis is generally consistent among fibrosis simulation studies, while the appearance of regular propagation patterns with augmented fibrosis is more controversial. Previous studies simulating diffuse fibrosis showed that the increase of fibrosis amount determined a transition from unstable to stable spirals via suppression of spiral wave break-up^37,38^. This process was explained in the light of a fibrosis-induced slowing of re-entrant waves with a shift of the system to a part of restitution curve with shallow slope, thus suppressing the steep-restitution spiral break-up^38^. Re-entrant wave period was similarly shown to increase superlinearly with the increase of fibrosis level and, despite irregular appearing of spatiotemporal wave patterns, the electrical activation was highly periodic and regular due to the instauration of a mother rotor^37^. In imaging-based atrial models, compact fibrosis was also shown to organize and stabilize re-entrant waves^23,31^. Rotors were anchored by proximal fibrotic regions and the enlargement of these regions decreased the frequency and widened the excitable gap of the re-entry, converting the fibrillatory process into an excitable gap re-entry^31^. Heterogeneous distributions of fibrosis slowed and stabilized rotors, which moved in the slow-conducting border zone surrounding inner regions of dense fibrosis^23^. In contrast with the described organizing effects, augmented pattern complexity, fragmentation into multiple wavefronts, and zig-zag propagation accompanied the reduction of conduction velocity and DF at higher fibrosis levels in an anatomically-realistic biatrial model^20^. Differences in results may be mainly related to the higher complexity of the model in^20^, which included realistic anatomies and fiber structures and modeled fibrosis in terms of cell-coupling with fibroblasts and myofibroblasts. Although we observed an overall organizing effect of fibrosis, higher variability and stochasticity of the propagation patterns and EGM-derived indices were present at high fibrosis density, especially for compact and patchy fibrosis elements (Figure S2). High stochasticity of simulation results and dependence of propagation patterns on the specific location of fibrotic cells was observed at higher fibrosis density in 2D ventricular lattice models with heterogeneous fibrosis distributions^37^. Non-uniform distribution of fibrosis, notwithstanding the identical overall fibrosis amount, substantially affected the arrhythmogenesis of patchy and compact fibrosis in anatomically-detailed ventricular models^40^. Our results showed no statistically-significant direct association between the frequency-domain indices and the size parameter *p*_*th*_ (Tables S3 and S4). This suggests that a single index of fibrotic element dimension may be insufficient to determine the emerging patterns, and a multiparametric description of fibrotic element distribution and clustering^36,40^ may be required to associate fibrosis patterns with specific arrhythmic mechanisms. In addition to fibrosis amount and size, we analyzed the effects of patchy fibrosis, mimicked by elongated fibrotic elements, on the propagation dynamics. Patchy fibrosis favored wave propagation along the channels determined by fibrosis and hindered propagation in the orthogonal direction, as testified by DCxy values, but it did not change the rate, regularity, or overall coupling, nor the stability of the patterns. Similar to our results, in a 2D lattice monolayer model, obstacle elongation produced anisotropic propagation and zig-zag conduction of planar wavefronts with longitudinal velocity increased and transverse direction decreased with elongation^39^. In a bilayer atrial model under pacing condition, stringy fibrosis produced prolongation and local fluctuation of activation times and increased anisotropy, but not zig-zag conduction as observed in monolayers, since 3D-like pathways were available for impulse propagation^34^. In a realistic 3D ventricular model^40^, patchy fibrosis exerted effects dependent on its spatial distribution and was associated with propagation patterns of intermediate complexity with respect to those produced by diffuse and compact fibrosis. In our study, the slowing and organization of impulse propagation at increased fibrosis amount led to a stabilization of the re-entrant activity, with a progressively higher fraction of simulated activity persisting along the 5 s observation window. Arrhythmia stabilization by fibrosis is consistent with simulation, experimental, and clinical findings^6,7^. Focusing on in-silico studies^26,40^, AF was induced and sustained only with higher degrees of fibrosis in anatomically-detailed atrial models^26^, and the likelihood of sustaining re-entry in 3D ventricular models^40^ was higher as the intensity of fibrosis increased independently of fibrosis texture.

Our study has several limitations, which hinders the direct transferability of our findings to the real AF case, where additional mechanisms may concur to re-entry formation and stability. First, the spherical geometry we adopted is an oversimplification of the atrial anatomy and does not reproduce its topology. In the real atrial anatomy, the presence of holes, associated with the pulmonary vein orifices and the valves, as well as atrial curvature differences, may concur to stabilize re-entrant waves, and the location of the fibrotic elements with respect to anatomic features may influence the patterns. The relative contribution of anatomical components and fibrosis in stabilizing the patterns may depend on fibrosis amount and the propagation pattern considered. Previous simulation studies^30^ showed that at higher degrees of fibrosis, the presence of large quantities of slow conducting fibrotic tissue facilitated the anchoring of re-entrant waves to the fibrotic patches, while at lower fibrosis burden re-entrant drivers moved near small fibrotic patches or drifted towards and anchored to the pulmonary veins and mitral valve annulus, due to the underlying atrial curvature. Moreover, the uncertainty in anatomy was more important for determining macro-re-entries and terminations, while uncertainty in fibrosis distribution was more important for functional re-entries, suggesting a specific role of the two factors in relation to different arrhythmic mechanisms^24^. Given these considerations, it is likely that our results, mostly relying on functional re-entries, may hold in a realistic geometry in the case of higher amounts of fibrosis, while at lower amounts, where our pattern was dominated by spiral break-up, the integration of anatomical structures may stabilize the re-entrant activity around an anatomical obstacle, organizing the activity at the local or global level. As a second limitation, we implemented a monolayer model with homogeneous conductivity values except for fibrosis, without including atrial thickness, nor fibre structure and conduction anisotropy. The presence of atrial wall thickness gradients per se, or in combination with fibrosis patterns, may create a 3D substrate for AF, attracting drivers and favouring the occurrence of intramural re-entries and breakthroughs. In the seminal experimental study by Zhao et al.^12^ in the isolated heart, AF re-entrant driver location correlated with specific structural characteristics, such as intermediate atrial wall thickness and fibrosis in regions with twisted transmural myofiber orientations. Simulation studies^32,59^ showed that the relative contribution to re-entry of atrial thickness and fibrosis differed in the two atrial chambers and in the presence of different fibrosis amounts, and that the two factors exerted competing influences in the drift and anchoring of AF re-entrant drivers. In a simplified slab model without fibrosis, re-entrant drivers drifted toward and then along steps of atrial wall thickness, due to the presence of a large source-to-sink mismatch in regions of high atrial wall thickness gradients^32^. The addition of fibrosis acted as a competing attractor for the re-entrant drivers, which localized in regions between the step and fibrosis. In realistic atrial anatomies and thickness distributions, both fibrosis and atrial thickness gradients affected re-entrant driver locations in the right atrium (RA), while in the left atrium (LA) locations were mainly determined by fibrosis or anatomical structures, due to the absence of large atrial wall thickness gradients^32^. Chamber specific effects of atrial wall thickness were confirmed in a recent study, which showed that AF driver regions were thicker and had more variable thickness than non-driver regions in the RA, while they were thinner than non-driver regions in the LA. The effects of fibre structure on conduction present also significant chamber dependence and interplay with fibrosis^33^. The RA and LA display significant differences in terms of fibre organization, both for gross structure and fibre architecture^60^. The RA is dominated by prominent gross structures, while the major part of the LA is smooth-walled, albeit with a multilayered and entangled fibre architecture and high inter-patient variability. Given the complexity of fibre architecture and the difficulty of clinical measurements in the single patient, recent studies^29,33^ proposed the use of isotropic conduction in LA models, based on the assumption that the composition of different fibre orientations across wall thickness and along the atrial surface produced an effective “isotropization” of propagation in the LA, which resulted in a low sensitivity of local activation times and gross propagation patterns to differences in fibre organization. The accuracy of LA activation maps produced by a fibre-independent isotropic models with tuned diffusion coefficients was proven high (i.e., 96% for focal and 93% for rotor arrhythmias) and consistent with a model incorporating myocardial fibres with the same geometry^29^. The dependence of AF driver location on atrial fibre field further decreased in the presence of fibrotic remodelling and electrical heterogeneity, when the micro re-entrant substrate became more spatially variegated^61^. These studies partially support the use of an isotropic monolayer in our study and the validity of our results to reproduce functional re-entry dynamics in the LA. However, the assumption of fully-transmural fibrosis, inherent to the monolayer, makes our model unsuitable for the analysis of 3D propagation phenomena. In the majority of simulation studies where atrial thickness was integrated, fibrosis was also assumed to be fully-transmural or transmurally-projected, while only a few recent studies evaluated 3D phenomena using bi or multi-layer simulation models, with layer-dependent microstructure, coupling, and fibrosis^12,25,34,35^. Different degrees of AF complexity, with different numbers of waves, breakthroughs and epi-endocardial dissociation, as observed in patients, were recently reproduced in a multilayer model, which included accurate fiber organization and different patterns of patchy or uniform fibrosis, located in the sole epicardial layer^25^. Generation of single-layer fibrosis patterns by our stochastic generator algorithm and integration in multilayer models may be pursued in future studies to extend our analysis to the 3D dynamics of re-entries.

## Conclusions

In this study we performed a systematic analysis of the effects of fibrosis geometrical features on the rhythmic properties and stability of re-entrant waves in an electrically-remodeled atrial tissue. We showed that the increase of fibrosis amount determined a progressive slowing and overall organization of the re-entrant waves, leading to an augmented stability. At the increase of fibrosis, the variety and stochastic nature of re-entrant patterns increased, especially in the presence of compact and patchy fibrosis. These results strengthen the evidence that fibrosis amount and spatial distribution play a crucial role in dictating re-entry dynamics. Further studies are needed to investigate how these mechanisms may interact with other proarrhythmic factors in supporting AF maintenance.

## Supplementary Information

Supplementary Information.

## Data Availability

The datasets generated and/or analyzed in the current study are available from the corresponding author on reasonable request.
