# Supplementary material for: Systematic in-silico evaluation of fibrosis effects on re-entrant wave dynamics in atrial tissue
Source: Sci Rep. 2024 May 19;14:11427. doi: 10.1038/s41598-024-62002-5 (PMC11639732; doi:10.1038/s41598-024-62002-5)

**Systematic in-silico evaluation of fibrosis effects**

**on re-entrant wave dynamics in atrial tissue**

**Supplementary Materials**

Masè M^1,*^, Cristoforetti A^1^, Pelloni S^1^, and Ravelli F^1^.

# ^1^ Laboratory of Biophysics and Translational Cardiology, Department of Cellular, Computational and Integrative Biology - CIBIO, University of Trento, 38123 Trento, Italy 2

# ^2^ CISMed - Centre for Medical Sciences, University of Trento, 38122 Trento, Italy

Running Title: Fibrosis and re-entrant waves in atrial tissue

*Corresponding author:

Michela Masè

Laboratory of Biophysics and Translational Cardiology,

Department of Cellular, Computational and Integrative Biology—CIBIO, University of Trento

Via Sommarive 18

38123 Povo-Trento, Italy

E-mail: [michela.mase@unitn.it](mailto:michela.mase@%20unitn.it)

**SUPPLEMENTARY METHODS**

**Description of the stochastic generation algorithm**

**Table S1.** Pseudocode of the stochastic generation algorithm. The iterative procedure randomly selects candidate nodes **n_cand_** from patch border (*I_border_*) or free regions and assigns them to the fibrotic node list (c(**n_cand_**)=1). Selection and assignment are based on the generation of two random numbers *q* and *r* and the fulfilment of two conditions, controlled by the input parameters *p_th_* and α. The iterative procedure is stopped when the temporary block density *D_temp_* reaches the input density *D*. To improve computation efficiency, the border list *I_border_* is updated only after a fraction (*f_update_*) of nodes has been successfully added to the patch.

While $D_{temp}<D$

Generate $q,r\in U\left[ 0 1 \right]$

If $q>p_{th}$

While $r\geq\left| {\hat{\mathbf{v}}}_{d}\cdot{\hat{\mathbf{v}}}_{b} \right|^{\alpha}$

Randomly select $\mathbf{n}_{cand}$ with $i\in I_{border}$

Compute ${\hat{\mathbf{v}}}_{b}$

If $r<\left| {\hat{\mathbf{v}}}_{d}\cdot{\hat{\mathbf{v}}}_{b} \right|^{\alpha}$

$$c\left( \mathbf{n}_{cand} \right)\to1$$

$N_{spent}\to N_{spent}+1$

If $\frac{N_{spent}}{size(I_{border})}\geq f_{update}$

Update $I_{border}$

$N_{spent}\to0$

End

End

End

Else

Randomly select $\mathbf{n}_{cand}$ with $c\left( \mathbf{n}_{cand} \right)=0$ and $cand\notin I_{border}$

$$c\left( \mathbf{n}_{cand} \right)\to1$$

Update $I_{border}$

$N_{spent}\to0$

End

Update $D_{temp}$

End

**Validation of the stochastic generation algorithm**

The stochastic generation algorithm was tested to assess the reliable generation of fibrotic patterns with different properties by tuning the input parameters (*D,* *p_th,_* α). Fibrotic pattern generation was first assessed in the isotropic condition (α=0), producing 100 realizations of the stochastic process for each combination of the *D* (3, 5, 12, 21, 30, 40) and *p_th_* (0.008, 0.01, 0.015, 0.03, 0.05, 0.07, 0.1) parameters (42 combinations). In each realization, the actually generated fibrotic density (*D_g_*) was calculated as the number of fibrotic nodes over total number of nodes in the mesh. The dimensions of fibrotic elements in each realization were characterized in terms of the weighted mean (*S_mean_*), standard deviation (*S_SD_*), and maximum size (*S_max_*) of the size distribution in each realization. The weight of each fibrotic element was defined as the number of nodes it contained. The use of weights limited the underestimation of the element size caused by the left-side of the size distribution (patches with a small number of nodes). Fibrotic pattern generation was then assessed at the change of the anisotropy parameter α. In this case, 100 realizations of the stochastic process were run for each combination of the α (0, 1, 2, 3, 4) and *p_th_* (0.008, 0.01, 0.015, 0.03, 0.05, 0.07, 0.1) parameters (35 combinations) at a density *D*=30%. In each realization, the distribution of angles *θ* (range [-π/2; π/2]), formed by the direction of the accepted border nodes, ${\hat{\mathbf{v}}}_{b}$, and the predefined anisotropy direction, ${\hat{\mathbf{v}}}_{d}$, (see Fig.1a in the main text), was computed. The mean value (*θ_mean_*) and standard deviation (*θ_SD_*) of the distribution were used as a surrogate measure of the anisotropy of the generated patterns, where narrow angle distributions peaking at null angle indicated marked anisotropy. For all extracted parameters, data were expressed as mean and standard deviation over the 100 realizations. In the case of angle values, circular statistics was used.

**Synthetic signal generation and analysis**

Simulated bipolar signals were generated during the observation window of each simulation and analyzed in the frequency-domain to provide a quantitative description of propagation pattern properties.

Bipolar EGMs were obtained using the current source approximation.^1^ The extracellular potential at a spatial point $\mathbf{P}$ was computed according to:

$\varphi\left( \mathbf{P} \right)=\sum_{k=1}^{N} \frac{I_{m}^{k}}{\left\| \mathbf{n}_{k}-\mathbf{P} \right\|}\Omega_{k}$ (Eq.1)

where the sum is extended to all the $k=1\ldots N$ nodes of the mesh, $I_{m}^{k}$ is the transmembrane current (i.e., sum of the current due to the membrane capacitance, ionic currents, and stimulus current) per unit area at node index $k$, $\mathbf{n}_{k}$ the position vector of the node, and $\Omega_{k}$ the surface area associated to the node. To obtain bipolar electrograms, virtual recording electrodes of 0.25 mm diameter were positioned at 0.5 mm distance from the sphere, on eighteen regularly spaced splines (π/9 rad angular distance), each composed by eight regularly spaced bipoles with 2 mm interelectrode distance. Bipolar electrograms were computed as the difference between the extracellular signals recorded at the two electrodes of each bipole. In each simulation, a set of 144 synthetic bipolar EGMs of 5 s length were acquired with a sampling frequency of 1 kHz. The first 500 ms were discarded to let the patterns stabilize and the remaining signal segments were analyzed. The peak-to-peak (P2P) voltage amplitude of each EGM was extracted and used as a correlate of fibrosis presence.^2^

A frequency-domain analysis framework was applied to extract the rhythmic properties of the EGMs, in terms of rate, regularity, and coupling metrics. Rate and regularity metrics were quantified in each EGM by quantifying the dominant frequency (DF) and regularity index (RI).^3,4^ Standard preprocessing steps, such as bandpass filtering between 40 and 250 Hz, rectification, and low-pass filtering with a 20-Hz cutoff, were applied to EGMs to optimize DF/RI quantification. A 4096-point Fast Fourier Transformation (FFT, spectral resolution 0.24 Hz) was applied for DF determination.

The DF of the spectrum, defined as the frequency of the maximal spectral amplitude, was automatically detected and assumed as the rate of the activation process. DF outliers were reviewed and corrected in the case of misdetections due to harmonics.

The regularity index (RI) was assumed as a measure of electrogram organization in terms of spectral dispersion. It was quantified as the ratio of the power of the DF (within 0.5 Hz) and the three following harmonics, over the total power (calculated from 2.5 Hz up to, but not including, the fifth harmonic peak):

$RI=\frac{\mathrm{Area}\left( DF+harmonics \right)}{Area below frequency spectrum}$ (Eq.2)

RI ranged between 0 and 1, with higher values indicating more organized electrical activity. DF estimates obtained from spectra with RI ≤ 0.2 were excluded from evaluation, being considered not reliable.^4^

The level of coupling of the electrical activity between neighboring bipoles (on the same spline, or between adjacent splines) was evaluated in terms of magnitude-squared coherence (MSC).^5,6^ Before the computation of MSC, EGMs were low-pass at 60 Hz (3-pole Butterworth) and down-sampled at 100 Hz. The MSC between two preprocessed EGMs, *x* and *y,* was computed as the ratio:

$$MSC(f)=\frac{\left| S_{xy}(f) \right|^{2}}{S_{xx}{\left( f \right)\cdot S}_{yy}(f)}$$

(Eq.3)

where *f* is the frequency, S_xy_ the cross-power spectrum, and S_xx_ and S_yy_ the individual power spectra of x and y. The MSC was estimated using an averaged FFT spectral estimate with overlapping window (128 points window with half-window overlapping). The coupling index Cxy of each pair of EGMs was estimated as the mean value of the MSC over the frequency range. Cxy ranged between 0 and 1, with 0 indicating the absence of coupling and 1 maximal coupling.

**Statistical analysis**

The association between EGM-based properties (average and variability values of P2P, DF, RI, Cxy, and DCxy) or pattern stability and the geometrical features of fibrosis was assessed using generalized linear models (GLM). To evaluate the effects of fibrosis amount and size, a GLM was separately fit to each EGM-based or stability index for the simulation subset with unoriented fibrotic elements (*α*=0). The index represented the dependent variable, while *D* and *p_th_* parameters were the independent factors. The effects of fibrosis orientation were similarly assessed by GLMs, restricting the analysis to the simulation subset with *D*=30%, in the absence (*α*=0) and presence of patchy/stringy fibrosis (α=4). In this case, the independent factors of the models were α and p_th_. A normal distribution with identity link function was used for EGM-based indices. A Poisson distribution with logistic link function was used for pattern stability, where the dependent variables was the binary class: stable (=1) or failed (=0) propagation. For all models, post-hoc comparisons were performed in the presence of statistically-significantly associations. P-values for model factors and in post-hoc comparisons were corrected for multiple comparisons using Bonferroni correction. A p-value < 0.05 was considered statistically significant.

**References**

1. Jacquemet, V. *et al.* Study of unipolar electrogram morphology in a computer model of atrial fibrillation. *J Cardiovasc Electrophysiol* **14**, S172-179 (2003).

2. Almeida, T. P. *et al.* Unsupervised Classification of Atrial Electrograms for Electroanatomic Mapping of Human Persistent Atrial Fibrillation. *IEEE Trans Biomed Eng* **68**, 1131–1141 (2021).

3. Everett, T. H., Kok, L. C., Vaughn, R. H., Moorman, J. R. & Haines, D. E. Frequency domain algorithm for quantifying atrial fibrillation organization to increase defibrillation efficacy. *IEEE Trans Biomed Eng* **48**, 969–978 (2001).

4. Ng, J., Kadish, A. H. & Goldberger, J. J. Effect of electrogram characteristics on the relationship of dominant frequency to atrial activation rate in atrial fibrillation. *Heart Rhythm* **3**, 1295–1305 (2006).

5. Ropella, K. M. Frequency domain analysis of endocardial signals. *Ann Ist Super Sanita* **37**, 351–359 (2001).

6. Ropella, K. M., Sahakian, A. V., Baerman, J. M. & Swiryn, S. The coherence spectrum. A quantitative discriminator of fibrillatory and nonfibrillatory cardiac rhythms. *Circulation* **80**, 112–119 (1989).

**SUPPLEMENTARY RESULTS**

**Results of the validation of the stochastic generation algorithm**

The response of the stochastic generation algorithm to changes in the parameter *D* and *p_th_* is shown in Figure S1 for the isotropic case (α=0). The algorithm was able to generate patterns with the predefined density (panel a) for all the size parameters, with density values that differed from the input values by an average bias from 0.0005 ± 0.0010% for D=3% to 0.07 ± 0.03 for D=40%. The tuning of the size parameter *p_th_* controlled the number and dimension of the generated patches (panels b to e). At the increase of *p_th_*, a progressive overall increase in the number of fibrotic elements (panel b, from 334 ± 210 at *p_th_* = 0.008 to 3554 ± 2005 at *p_th_* = 0.1) and decrease in their mean size (panel c, from 918 ± 162 nodes at *p_th_* = 0.008 to 59 ± 11 nodes at *p_th_* = 0.1) were observed for all fibrosis densities. The decrease of the element mean size was accompanied by a decrease in the maximal size (panel d, from 3488 ± 1276 nodes at *p_th_* = 0.008 to 443 ± 158 nodes at *p_th_* = 0.1) and in the dispersion of the size distribution (panel e, from 984 ± 273 at *p_th_* = 0.008 to 74 ± 16 at *p_th_* = 0.1). In terms of density parameter *D*, the number of fibrotic elements and all size parameters progressively increased at the increase of *D*. Differences in the number of fibrotic elements between density levels were more marked for larger *p_th_* values (i.e., for smaller patch sizes; at *p_th_* = 0.1, the number of fibrotic elements was 838 ± 24 for D=3% and 6120 ± 73 for D=40%). Differences in size were larger for smaller *p_th_* values (i.e., for larger patch sizes) and more marked for the maximal size and dispersion (at *p_th_* = 0.008, *S_max_* = 2001 ± 434 nodes and *S_SD_* = 742 ± 214 nodes for D=3%, and *S_max_* = 4878 ± 986 nodes and *S_SD_* = 1187 ± 230 nodes for D=40%) than for the mean size (at *p_th_* = 0.008, *S_mean_* = 831 ± 209 nodes for D=3%, and 1014 ± 102 nodes for D=40%).

The control exerted by the anisotropy parameter α on the accepted angles θ is exemplified in the upper panels of Figure 2S. The increase of α from 0 to 4 (panels a to c) was accompanied by a progressive restriction of the range of accepted angles. In the isotropic case (α=0) the angles presented an almost uniform distribution (*θ_mean_*= -1.54 rad and *θ_SD_* 1.53 rad), indicating no restriction on the accepted angles. Conversely, restrictions on the accepted angles became more stringent at the increase of the parameter α, with angle distributions presenting peaks close to the null angle (*θ_mean_* = 0.015 rad and 0.001 rad in panels b and c, respectively) and progressively smaller dispersion (*θ_SD_* = 0.75 rad and 0.57 rad in panels b and c, respectively). Overall results on the 100 realizations of the stochastic process, shown in the bottom panels, confirmed these trends. The mean angle shifted to null values for α>0 (panel d) and the dispersion progressively decreased from *θ_SD_* =1.47 ± 0.05 rad at α=0 to 0.58 ± 0.01 rad at α=4 (panel e) without marked differences for different *p_th_* values.

**Figure S1.** Control of the stochastic generation algorithm of fibrotic patterns by the density (*D*) and size (*p_th_*) parameters in the isotropic case (α=0). Generated fibrosis density *D_g_* as a function of the set input parameter *D,* color-coded for different *p_th_* values (a). Number of generated fibrosis elements (b), weighted mean (*S_mean_*, c), maximum (*S_max_*, d), and standard deviation (*S_SD_*, e) of the element size distributions (e) as a function of the size parameter *p_th_*. Patterns obtained for different densities are indicated in colour scale. In all panels, dots and whiskers indicate mean and standard deviation over the 100 realizations of the stochastic process.


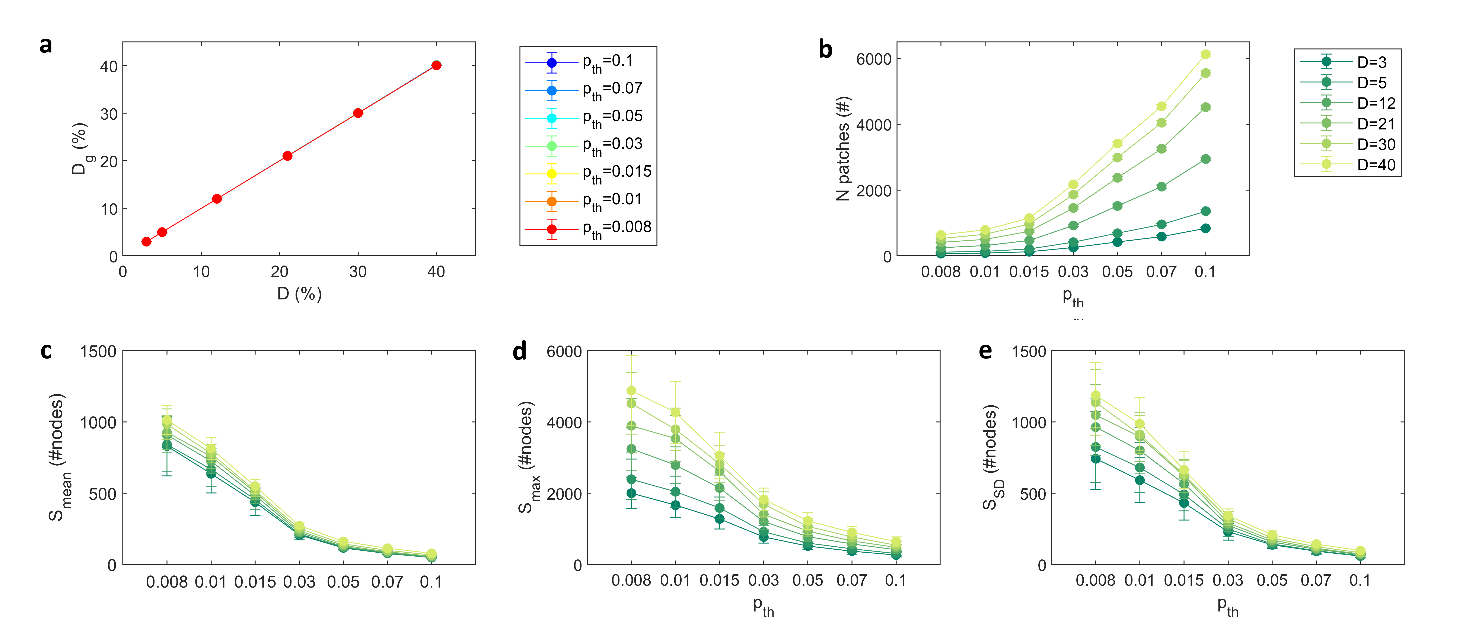


**Figure S2.** Control of the anisotropic growth of the fibrotic patches by the anisotropy parameter α. Upper panels. Representative distributions of the angle *θ,* formed by the direction of accepted border nodes, ${\hat{\mathbf{v}}}_{b},$ and the predefined direction, ${\hat{\mathbf{v}}}_{d}$, at increasing values of α. In the isotropic case (α=0, in panel a), all directions are accepted, and the distribution is uniform, while at increasing values of α (panels b and c), nodes aligned to ${\hat{\mathbf{v}}}_{d}$ are preferentially selected and the distributions become narrower and peaked on the null angle. Lower panels. Mean value (*θ_mean_* in panel d) and standard deviation (*θ_SD_* in panel e) of the angle distributions obtained at each realization of the stochastic process, as a function of the anisotropy parameter α. Errors bars indicate mean and standard deviation on 100 realizations of the stochastic process and are color-coded according to the size parameter *p_th_*. Note the shift toward the null angle and the progressive decrease in the dispersion of the angle distribution at the increase of parameter α.

**
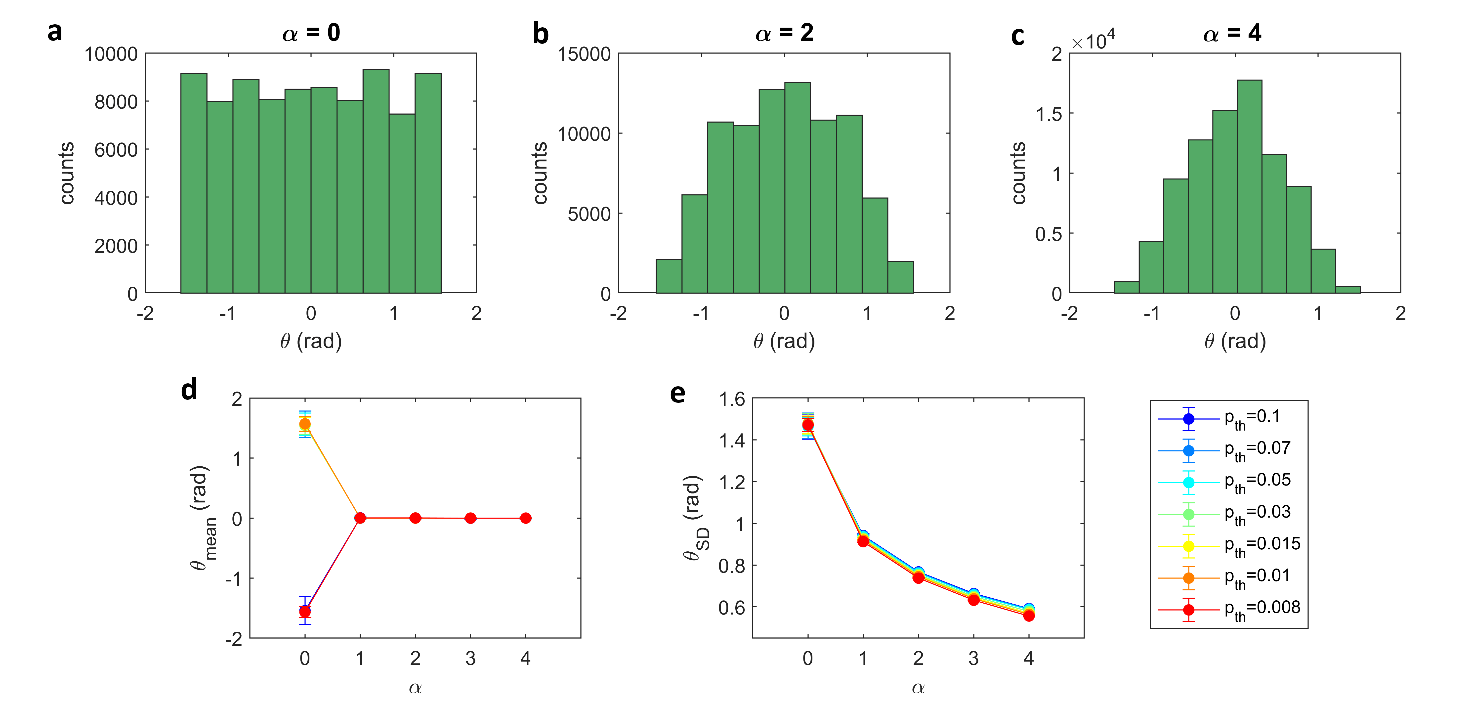
**

**Results of the performed simulations**

**Table S2.** Stability and electrogram-based indices of re-entrant patterns arising at different fibrosis amounts (density, D). Data are reported as number (percentage) or mean and standard deviation over the simulation subset at fixed D. Data include simulations with diffuse and compact fibrosis (unoriented elements, D=3% to D=40%) and patchy fibrosis (oriented elements, D=30or). Stability was computed on the whole simulation set (N=245), while electrogram-based indices only on simulation with stable propagation patterns (N=159).

|  | **D=3%** | **D=5%** | **D=12%** | **D=21%** | **D=30%** | **D=40%** | **D=30or** |
| --- | --- | --- | --- | --- | --- | --- | --- |
| **Stability,**  **N (%)** | 10 (28.6) | 16 (45.7) | 15 (42.9) | 25 (71.4) | 30 (86.7) | 29 (82.9) | 34 (97.1) |
| **P2P (au)** | 0.165±0.004 | 0.160±0.002 | 0.148±0.008 | 0.128±0.006 | 0.112±0.009 | 0.090±0.009 | 0.108±0.011 |
| **P2P_std_ (au)** | 0.033±0.003 | 0.037±0.004 | 0.047±0.007 | 0.055±0.007 | 0.060±0.009 | 0.058±0.011 | 0.054±0.010 |
| **DF (Hz)** | 4.34 ± 0.03 | 4.35 ± 0.07 | 4.29 ± 0.05 | 4.21 ± 0.10 | 4.07 ± 0.22 | 3.96 ± 0.22 | 4.07 ± 0.15 |
| **DF_std_ (Hz)** | 0.16 ± 0.02 | 0.15 ± 0.04 | 0.13 ± 0.03 | 0.10 ± 0.06 | 0.05 ± 0.06 | 0.07 ± 0.07 | 0.09 ± 0.07 |
| **RI (nu)** | 0.47 ± 0.04 | 0.47 ± 0.04 | 0.55 ± 0.08 | 0.68 ± 0.13 | 0.81 ± 0.13 | 0.91 ±0.09 | 0.82 ± 0.11 |
| **RI_std_ (nu)** | 0.12 ± 0.02 | 0.11 ± 0.02 | 0.13 ± 0.02 | 0.12 ± 0.04 | 0.09 ± 0.04 | 0.08 ± 0.04 | 0.11 ± 0.05 |
| **Cxy (nu)** | 0.27 ± 0.01 | 0.27 ±0.02 | 0.30 ± 0.03 | 0.38 ± 0.08 | 0.50 ± 0.14 | 0.56 ±0.09 | 0.46 ± 0.10 |
| **Cxy_std_ (nu)** | 0.09 ± 0.01 | 0.09 ± 0.01 | 0.11 ± 0.02 | 0.14 ± 0.02 | 0.15 ±0.03 | 0.16 ± 0.02 | 0.16 ± 0.01 |
| **DCxy (au)** | 1.08 ± 0.06 | 1.10 ± 0.05 | 1.12 ± 0.09 | 1.08 ± 0.06 | 1.06 ± 0.05 | 1.04 ± 0.05 | 0.91 ± 0.05 |
| **DCxy_std_ (au)** | 0.68 ± 0.07 | 0.69 ± 0.09 | 0.80 ± 0.10 | 0.82 ± 0.13 | 0.69 ± 0.24 | 0.61 ± 0.18 | 0.65 ± 0.11 |

Electrogram-based indices correspond to mean value and spatial variability (standard deviation, std subscript) over the electrode matrix of voltage amplitude (P2P), dominant frequency (DF), regularity index (RI), coupling (Cxy), and coupling asymmetry index (DCxy).

**Table S3.** Effects of fibrosis amount (density, D) and size parameter (p_th_) on pattern stability and electrogram properties. The reported values indicate the statistical significance (p-values corrected with Bonferroni correction) of intercept and fibrosis parameters D and p_th_, obtained by fitting separate generalized linear models to stability and electrogram-derived indices. For statistically-significant values, indicated in bold character, the results of statistically-significant post-hoc pairwise comparisons (p-values corrected with Bonferroni correction) are also indicated. Only simulations with diffuse and compact fibrosis (non-oriented elements, α=0) and presenting sustained propagation during the observation period (N=124) were considered.

|  | **Intercept** | **D** | **p_th_** |
| --- | --- | --- | --- |
| **Stability** | 0.06 | **<0.00001**  Post-hoc comparisons:  D=40% ^***,#,^^  D=30% ^***,#,^^^  D=21% ^**^ | 1.00 |
| **P2P** | **<0.00001** | **<0.00001**  Post-hoc comparisons:  D=40% ^***,###,^^^,$$$,£££^  D=30% ^***,###,^^^,$$$^  D=21% ^***,###,^^^^  D=12% ^***,###^ | **<0.00001**  Post-hoc comparisons:  p_th_=0.1 ^***,###,^^^,$$,&^  p_th_=0.07 ^***,###,^^^^  p_th_=0.05 ^***,###,^^^^  p_th_=0.03 ^**,###,^^^^ |
| **P2P_std_** | **<0.00001** | **<0.00001**  Post-hoc comparisons:  D=40% ^***,###,^^^^  D=30% ^***,###,^^^,$$^  D=21% ^***,###,^^^^  D=12% ^***,###^ | **<0.00001**  Post-hoc comparisons:  p_th_=0.1 ^***,###,^^^,$$$,£££^  p_th_=0.07 ^***,###,^^^,$$$^  p_th_=0.05 ^***,###,^^^^  p_th_=0.03 ^***,###,^^  p_th_=0.015 ^*,#^ |
| **DF** | **<0.00001** | **<0.00001**  Post-hoc comparisons:  D=40% ^***,###,^^^,$$$,£^  D=30% ^***,###,^^^,$$^ | 1.00 |
| **DF_std_** | **<0.00001** | **<0.00001**  Post-hoc comparisons:  D=40% ^***,###,^^^  D=30% ^***,###,^^^,$$^  D=21% ^*,##^ | 1.00 |
| **RI** | **<0.00001** | **<0.00001**  Post-hoc comparisons:  D=40% ^***,###,^^^,$$$,££^  D=30% ^***,###,^^^,$$$^  D=21% ^***,###,^^^ | 1.00 |
| **RI_std_** | **<0.00001** | **<0.00001**  Post-hoc comparisons:  D=40% ^*,##,^^^,$$$^  D=30% ^^^,$^ | 1.00 |
| **Cxy** | **<0.00001** | **<0.00001**  Post-hoc comparisons:  D=40% ^***,###,^^^,$$$^  D=30% ^***,###,^^^,$$$^  D=21%, ^*,##^ | 1.00 |
| **Cxy_std_** | **<0.00001** | **<0.00001**  Post-hoc comparisons:  D=40% ^***,###,^^^^  D=30% ^***,###,^^^^  D=21% ^***,###,^^^^  D=12% ^#^ | 1.00 |
| **DCxy** | **<0.00001** | **<0.00001**  Post-hoc comparisons:  D=40% ^##,^^^^  D=30% ^^^^ | 0.38 |
| **DCxy_std_** | **<0.00001** | **<0.00001**  Post-hoc comparisons:  D=40% ^^^,$$$^  D=30% ^$^  D=21% ^#^ | 1.00 |

Electrogram-based indices correspond to mean value and spatial variability (standard deviation, std subscript) over the electrode matrix of voltage amplitude (P2P), dominant frequency (DF), regularity index (RI), coupling (Cxy), and coupling asymmetry index (DCxy).

In post-hoc pairwise comparisons among D subgroups, symbols correspond to:

^*^, ^**^, ^***^: p<0.05, p<0.01, p<0.001 vs D=3%; ^#^, ^##^, ^###^: p<0.05, p<0.01, p<0.001 vs D=5%;

^^^, ^^^^, ^^^^^: p<0.05, p<0.01, p<0.001 vs D=12%; ^$^, ^$$^, ^$$$^: p<0.05, p<0.01, p<0.001 vs D=21%;

^£^, ^££^, ^£££^, p<0.05, p<0.01, p<0.001 vs D=30%.

In post-hoc pairwise comparisons among p_th_ subgroups, symbols correspond to:

^*^, ^**^, ^***^: p<0.05, p<0.01, p<0.001 vs p_th_=0.008; ^#^, ^##^, ^###^: p<0.05, p<0.01, p<0.001 vs p_th_=0.01;

^^^, ^^^^, ^^^^^: p<0.05, p<0.01, p<0.001 vs p_th_=0.015; ^$^, ^$$^, ^$$$^: p<0.05, p<0.01, p<0.001 vs p_th_=0.03;

^£^, ^££^, ^£££^, p<0.05, p<0.01, p<0.001 vs p_th_=0.05; ^&^, ^&&^, ^&&&^, p<0.05, p<0.01, p<0.001 vs p_th_=0.07.

**Table S4.** Effects of fibrosis orientation (α) and size parameter (p_th_) on pattern stability and electrogram properties. The reported values indicate the statistical significance (p-values corrected with Bonferroni correction) of intercept and fibrosis parameters α and p_th_, obtained by fitting separate generalized linear models to stability and electrogram-derived indices. Only simulations with a fibrosis density of 30%, non-oriented (α=0, D=30%) and oriented elements (α=4, D=30or), and presenting sustained propagation during the observation period (N=64 simulations) were considered. Statistically-significant values are reported in bold character. For statistically-significant associations with p_th_, post-hoc pairwise comparisons (p-values corrected with Bonferroni correction) are also indicated.

|  | **Intercept** | **α** | **p_th_** |
| --- | --- | --- | --- |
| **Stability** | 1.00 | 1.00 | 1.00 |
| **P2P** | **<0.00001** | 1.00 | **<0.00001**  Post-hoc comparisons:  p_th_=0.1 ^***,###,^^^,$$^  p_th_=0.07 ^***,###,^^^^  p_th_=0.05 ^***,###,^^^^  p_th_=0.03 ^***,###,^^^  p_th_=0.015 ^##^ |
| **P2P_std_** | **<0.00001** | **<0.0005** | **<0.00001**  Post-hoc comparisons:  p_th_=0.1 ^***,###,^^^,$$$,£££^  p_th_=0.07 ^***,###,^^^,$$$^  p_th_=0.05 ^***,###,^^^,$$^  p_th_=0.03 ^***,###,^^^^  p_th_=0.015 ^**^ |
| **DF** | **<0.00001** | 1.00 | 1.00 |
| **DF_std_** | **<0.00001** | 0.26 | 1.00 |
| **RI** | **<0.00001** | 1.00 | 1.00 |
| **RI_std_** | **<0.00001** | 1.00 | 0.55 |
| **Cxy** | **<0.00001** | 1.00 | 0.54 |
| **Cxy_std_** | **<0.00001** | 0.45 | 1.00 |
| **DCxy** | **<0.00001** | **<0.00001** | 1.00 |
| **DCxy_std_** | **<0.00001** | 1.00 | 1.00 |

Electrogram-based indices correspond to mean value and spatial variability (standard deviation, std subscript) over the electrode matrix of voltage amplitude (P2P), dominant frequency (DF), regularity index (RI), coupling (Cxy), and coupling asymmetry index (DCxy).

In post-hoc pairwise comparisons among p_th_ subgroups, symbols correspond to:

^*^, ^**^, ^***^: p<0.05, p<0.01, p<0.001 vs p_th_=0.008; ^#^, ^##^, ^###^: p<0.05, p<0.01, p<0.001 vs p_th_=0.01;

^^^, ^^^^, ^^^^^: p<0.05, p<0.01, p<0.001 vs p_th_=0.015; ^$^, ^$$^, ^$$$^: p<0.05, p<0.01, p<0.001 vs p_th_=0.03;

^£^, ^££^, ^£££^, p<0.05, p<0.01, p<0.001 vs p_th_=0.05; ^&^, ^&&^, ^&&&^, p<0.05, p<0.01, p<0.001 vs p_th_=0.07.

**Figure S3.** Electrogram-based indices for different fibrosis distributions. Average value (top panels) and spatial variability (std subscript, bottom panels) of voltage amplitude (P2P and P2P_std_), rate (DF and DF_std_), regularity (RI and RI_std_), and coupling (Cxy and Cxy_std_) are shown as a function of the size parameter (p_th._) and color-coded according to fibrosis overall amount (density, D) and orientation. Data are calculated on simulations with stable propagation patterns (N=159), with diffuse and compact fibrosis (unoriented elements, D=3% to D=40%) and patchy fibrosis (oriented elements, D=30or). Black dots and whiskers indicate average values and standard deviation over simulation subgroups, while small colored dots correspond to single simulation outcomes.

**
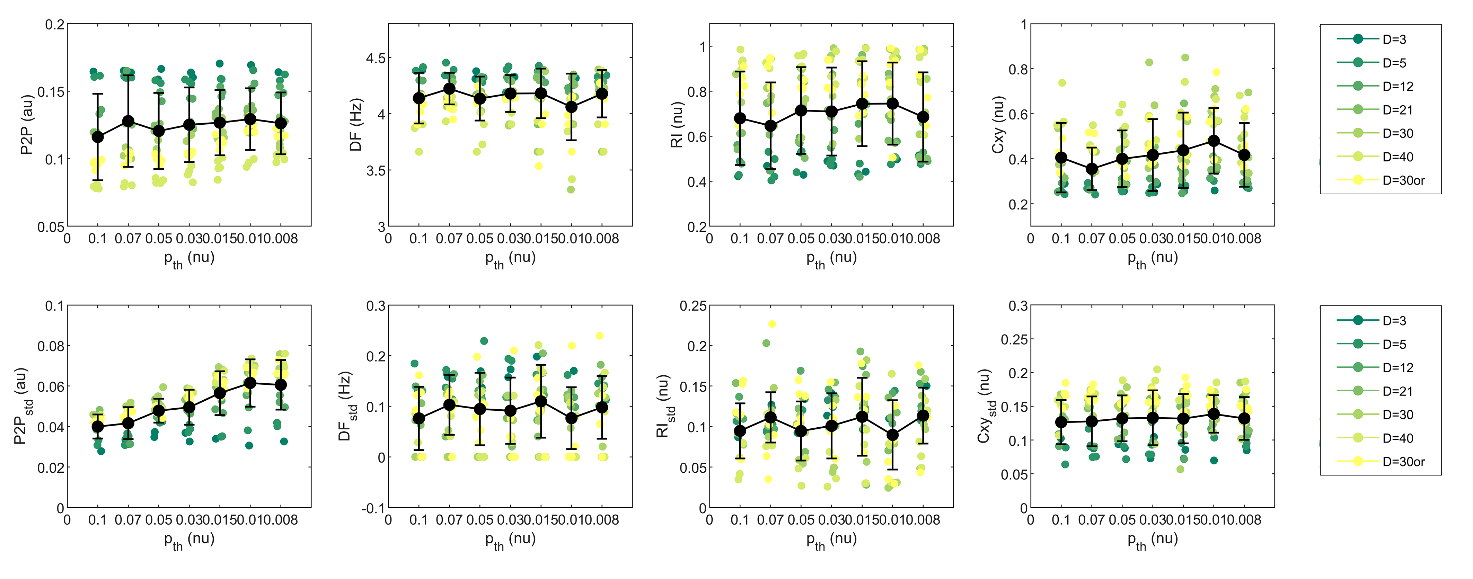
**

**Figure S4.** Variability of electrogram-derived indices over five repetitions at fixed fibrosis parameters. The variability is quantified as standard deviations over five repetitions and displayed as a function of the fibrosis size parameter (p_th_). Different lines and colors correspond to different overall amounts of fibrosis (density, D) and orientations. Data are calculated on simulations with stable simulation patterns (N=159), with diffuse and compact fibrosis (unoriented elements, D=3% to D=40%) and patchy fibrosis (oriented elements, D=30or). Note that higher variability/stochasticity of the simulation outcomes are observable for higher amount of fibrosis and smaller p_th_ values (i.e., for larger element sizes).


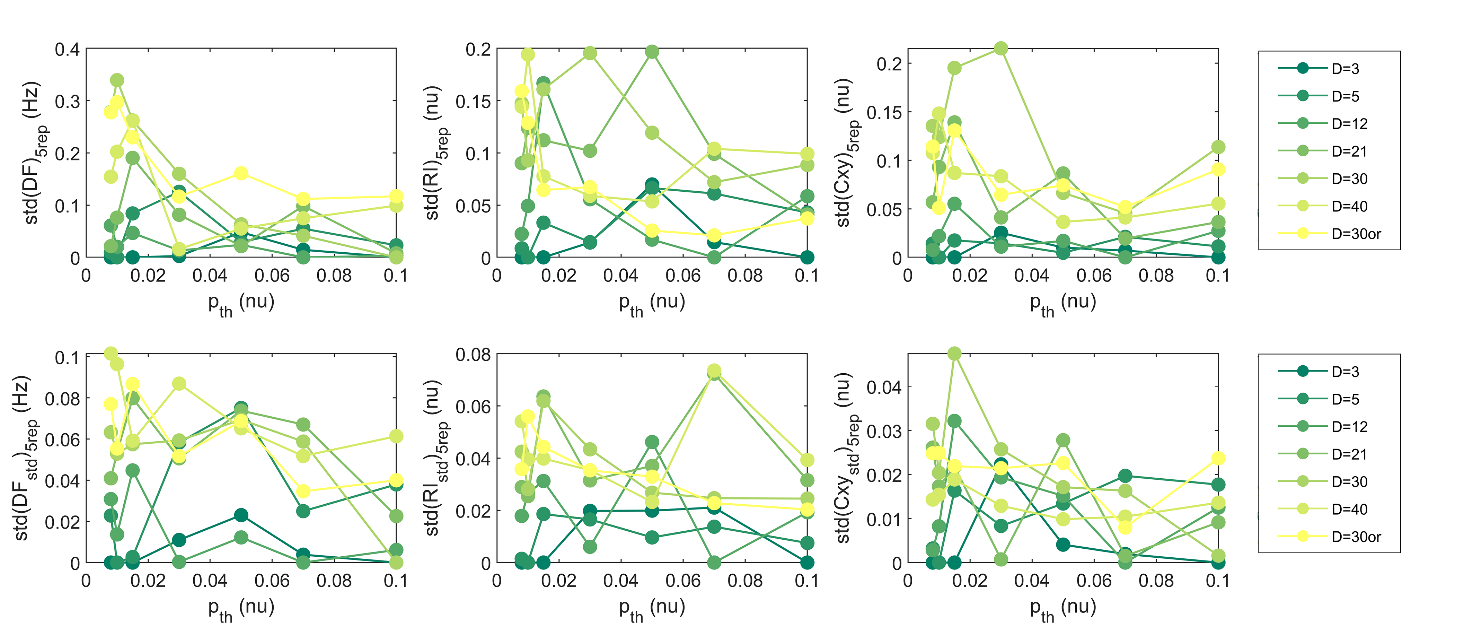

Supplement: Supplementary file 1 — Supplementary Information. [file 41598_2024_62002_MOESM1_ESM.docx]
